# De novo assembly of *plasmodium interspersed repeat* (*pir*) genes from *Plasmodium vivax* RNAseq data suggests geographic conservation of sub-family transcription

**DOI:** 10.1186/s12864-025-11752-1

**Published:** 2025-05-29

**Authors:** Timothy S. Little, Deirdre A. Cunningham, George K. Christophides, Adam James Reid, Jean Langhorne

**Affiliations:** 1 The Francis Crick Institute, Midland Road, London, UK; 2 Department of Life Sciences, Imperial College London, South Kensington, London, UK; 3 The Gurdon Institute, University of Cambridge, Tennis Court Road, Cambridge, CB2 1QN UK; 4 Present Address: UCL Respiratory, Rayne Building, University College London, London, UK

**Keywords:** Malaria, Vivax, Transcriptomics, Pir, Multigene

## Abstract

**Background:**

The *plasmodium interspersed repeats* (*pir*) multigene family is found across malaria parasite genomes, first discovered in the human-infecting species *Plasmodium vivax*, where they were initially named the *vir*s. Their function remains unknown, although studies have suggested a role in virulence of the asexual blood stages. Sub-families of the *P. vivax pir/vir*s have been identified, and are found in isolates from across the world, however their transcription at different localities and in different stages of the life cycle have not been quantified. Multiple transcriptomic studies of the parasite have been conducted, but many map the *pir* reads to existing reference genomes (as part of standard bioinformatic practice), which may miss members of the multigene family due to its inherent variability. This obscures our understanding of how the *pir* sub-families in *P. vivax* may be contributing to human/vector infection.

**Results:**

To overcome the issue of hidden *pir* diversity from utilising a reference genome, we employed de novo transcriptome assembly to construct the *pir* ‘reference’ of different parasite isolates from published and novel RNAseq datasets. For this purpose, a pipeline was written in Nextflow, and first tested on data from the rodent-infecting *P. c. chabaudi* parasite to ascertain its efficacy on a sample with a full, genome-based set of *pir* gene sequences. The pipeline assembled hundreds of *pir*s from the studies included. By performing BLAST sequence identity comparisons with reference genome *pir*s (including *P. vivax* and related species) we found a clustered network of transcripts which corresponded well with prior sub-family annotations, albeit requiring some updated nomenclature. Mapping the RNAseq datasets to the de novo transcriptome references revealed that the transcription of these updated *pir* gene sub-families is generally consistent across the different geographical regions. From this transcriptional quantification, a time course of mosquito bloodmeals (after feeding on an infected patient) highlighted the first evidence of ookinete stage *pir* transcription in a human-infective malaria parasite.

**Conclusions:**

De novo transcriptome assembly is a valuable tool for understanding highly variable multigene families from *Plasmodium spp*., and with pipeline software these can be applied more easily and at scale. Despite a global distribution, *P. vivax* has a conserved *pir* sub-family structure—both in terms of genome copy number and transcription. We suggest that this indicates important roles of the distinct sub-families, or a genetic mechanism maintaining their preservation. Furthermore, a burst of *pir* transcription in the mosquito stages of development is the first glint of ookinete *pir* expression for a human-infective malaria parasite, suggesting a role for the gene family at a new stage of the lifecycle.

**Supplementary Information:**

The online version contains supplementary material available at 10.1186/s12864-025-11752-1.

## Introduction

*Plasmodium vivax* is the most widely distributed species of *Plasmodium* that infects humans, causing recurrent malaria. Although globally the proportion of malaria cases attributable to *P. vivax* has been decreasing since 2000, *P. vivax* still accounts for approximately 50–80% of all human malaria cases in the Americas and most of Asia [1]. It has traditionally been thought that *P. vivax* was not a major cause of malaria in sub-Saharan African, however increasing evidence of seropositivity in African nations have led some to suggest that *P. vivax* in the continent is more endemic than common mantra suggests [2–4]. Given its prevalence around the world, *P. vivax* is a major focus of public health research.

A feature of the genome of all *Plasmodium* species is the presence of large multigene families [5]. One of most expansive of these is the *Plasmodium interspersed repeats* (*pir*s) family, which is present across the *Plasmodium* lineage [6]. This includes human-infective parasites such as *P. vivax*, *P. malariae* and *P. ovale*, simian-infective parasites such as *P. cynomolgi,* and the rodent-infecting *Plasmodium* species [6–8]. Sometimes the gene family is named differently depending on the species, such as the *vir*s in *P. vivax* or *cir*s in *P. c. chabaudi*, but they are all members of the *pir* gene family and are hereby referred to only as *pir*s. However, *pir* genes are not found in species within the subgenus Laverania (such as *P. falciparum*). Unlike the *var* genes of *P. falciparum*, which are known to play a role in sequestration in host endothelium [9] and contribute to severe pathology [10], relatively little is known about the exact function or binding partners of *pir* genes. However, we have previously shown that they are associated with virulence and establishment of chronic infection in rodent-infecting species [11, 12], and others have demonstrated that the surface PIR protein is involved in infected red blood cell sequestration [13, 14].

*P. ovale, P. malariae* and *P. yoelii* possess over 1000 *pir* members in their genomes, although for other species the copy number can be much fewer, such as the 134 *pir*s identified in the rodent malaria parasite *P*. *berghei* [15–18]*.* The *P. vivax* P01 strain reference genome contains 1216 members of the *pir* gene family [19], and a recent genome assembly of Thai-origin W1 contains as many as 1145 predicted *pir* genes [20], thus constituting around a sixth of the total number of predicted genes for this organism. Using sequence clustering and phylogenomics, one can further divide this multigene family into sub-families, one clade of sub-families specific to the rodent-infective species, and another specific to simian/human-infective species [16]. A prominent exception to this is a relatively highly conserved *pir* sequence postulated to be the ancestral gene, which is found across in the genomes of the rodent-infective and simian-infective clades of *Plasmodium sp.* [21]*.* From *P. vivax* genomes sequenced across different geographic regions, it has been shown that there are differences not just in the *pir* repertoire of these isolates, but also in the proportions of *pir* sub-family members around the world [22, 23]. The dedication of a large part of the *P. vivax* genome to a single multigene family suggests an important role, and understanding this is of high importance.

Although experimental *P. chabaudi* and *P. berghei* infections in mice provide ready access to RNA for studying the transcriptional patterns of the *pir* genes, human malarias, such as *P. vivax*, remain more challenging to investigate. Among the malaria parasite species, *P. vivax* genomes are particularly diverse between isolates, and this is heavily concentrated within multigene families such as the *pir*s [24].This presents an obstacle for transcriptomic studies, as *pir*s may be missed when mapping divergent isolates to a generic reference genome.

One approach to obtain a more comprehensive overview of the number and variability of *pir* genes in *P. vivax* is to use de novo transcriptomic assembly on the available *P. vivax* RNAseq data, a method in which the sequenced reads are used for assembly of the RNA being transcribed, without need of a reference genome. De novo transcriptome assembly has previously been performed for *Plasmodium* species with missing or inadequate reference genomes, including the assembly of *pir* gene transcripts. For example, novel *pir* genes were identified in a *P. yoelii nigeriensis* de novo transcriptomic assembly [25]. Similarly, *P. ovale* and a limited number of *P. vivax* studies [26–28] assembled novel transcripts which were annotated as *pir*-like. All these studies demonstrated marked differences between the *pir* repertoires of the reference genomes and the de novo transcriptomes, suggesting that, without the new assemblies, some *pir*s would have been completely missed. Using de novo transcriptome assembly permits the identification of novel members of highly variable multigene families and assessment of their levels of expression during different life stages and environments, ultimately enabling deeper investigation into their functions.

Here, we have analysed the transcription of *pir*s in published datasets of *P. vivax* RNAseq (see Table 1), using de novo transcriptome assembly to unlock a greater repertoire of the genes than those already existing in the reference genomes. We determined whether the pre-existing *pir* sub-family definitions accurately describe clusters of the de novo*-*generated genes. Once annotated, we questioned whether distinct sub-families are differently expressed across the parasite lifecycle and whether they vary between geographical locations. We compare the *pir* expression patterns between this human malaria parasite and rodent malaria species, which could suggest that the *pir* family has similar function between divergent species.

**Table 1:** Summary of the publications from which *P. vivax* RNAseq data was taken and used for de novo assembly. The study reference, life cycle stages/conditions extracted, geographic source of the isolates and experimental code used in this paper are all included. A descriptive short identifier was given to each experiment/dataset, stating the life cycle stages covered (e.g. ‘Asex’ for ‘Asexual stages’, ‘Mix’ if multiple stages are included), the country of origin (e.g.’Camb’ for ‘Cambodia', ‘Mix’ if multiple geographical sources are involved), and year of publication. Note that the ‘Asexual’ stages have not been explicitly depleted for sexual stages such as gametocytes or sexual-lineage schizonts, however these should be a minor contaminant if present

Reference	Life cycle stages	Strains used	Experiment code
Zhu et al., 2016 [28]	IDC (different lengths of time in ex vivo culture)	NW Thai patient isolates (Mae Sot)	AsexThai16
Kim et al., 2017 [27]	Mixed blood stages (and one sporozoite)	Ratanakiri, Cambodia	MixdCamb17 (Same authors as MixdCamb19)
Gural et al., 2018 [29]	Mixed liver stages and ‘hypnozoite-enriched’ (in vitro human organelle system)	Thai patient isolates	LivrThai18
Roth et al., 2018 [30]	Sporozoites	Myanmar border, Thailand	SpztThai18
Cheng et al., 2019 [31]	Asexual blood stages	5 N Thai and 5 S Thai patient isolates	AsexThai19
Muller et al., 2019 [32]	Sporozoites	Thai patient isolates (Tak/Ubon)	SpztThai19
Kim et al., 2019 [33]	Mixed blood stages (with differential counts)	26 Ratanakiri, Cambodia patient isolates	MixdCamb19 (Same authors as MixdCamb17)
Gunalan et al., 2019 [34]	Asexual blood stages from two species of monkey	*P. vivax* Sal I (El Salvador origin [35])	AsexSal119
Boonkaew et al., 2020 [36]	Ookinete (18 h) and Oocyst (7 days)	Thai patient isolates (Ubon and Yala)	MosqThai20
Siegal et al., 2020 [37]	Schizonts	Cambodia patient isolates	SchzMixd20
Rangel et al., 2020 [38]	IDC (4, 20, 36, 44 and 72 h post-thaw)	Acre, Brazil	AsexBraz20
Bourgard et al., 2021 [39]	Asexual blood stages	Manaus, Brazil	AsexBraz21
de Meulenaere et al., 2022 [40]	Schizonts	Iquitos, Peru, and Madang, Papua New Guinea	SchzMixd22
Carlos et al., in preparation	Mosquito blood-meal/midguts (1 h, 6 h, 22 h, 26 h, and 7 d)	Iquitos, Peru	MosqPeru23

## Results

### De novo transcriptome assembly generates full-length *pir* genes from samples of *Plasmodium chabaudi* blood stages

**Fig. 1:**
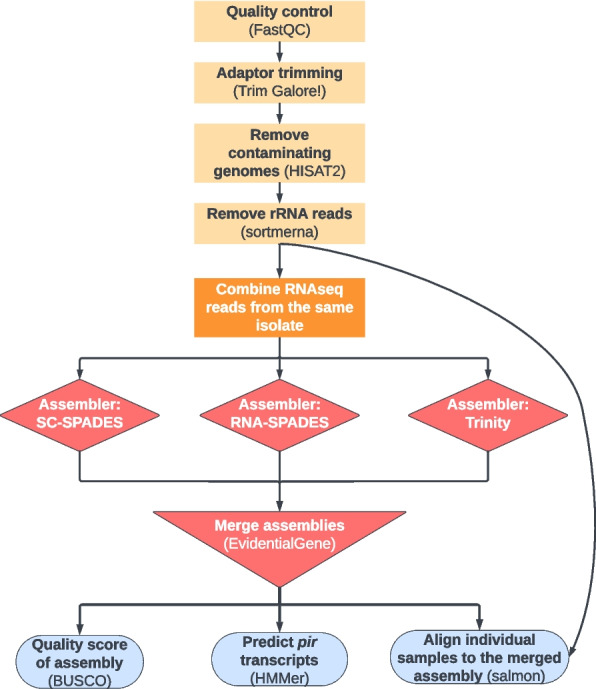
Workflow diagram of the pipeline used for generating the de novo transcriptome assemblies, predicting *pir* transcripts, and then obtaining expression data in TPM. Software used for each step is shown in brackets. Individual samples were concatenated together for the assembly process if they originate from the same source isolate (orange central box), however they were then aligned separately to the merged assembly (right-hand curved arrow) to see how expression changed by condition. Drawn using LucidPlot

In order to assemble the de novo transcripts of *P. vivax pir* genes it was first necessary to determine the most effective assembly method. For this, three different assemblers were used on RNAseq datasets from published *P. chabaudi chabaudi* AS RNAseq reads [41].These were then combined to identify the best transcripts using EvidentialGene [42, 43] (Fig. 1), assessing the outputs for both the number of known *pir* genes recovered and the number of conserved *Plasmodium* genes found among the transcripts (Benchmarking Universal Single-Copy Orthologs—BUSCO – a score of assembly quality [44]). There is no agreed threshold for a ‘good’ BUSCO score, so we aimed to get the highest scores that the data and software could produce. Among the individual tools, Spades-RNA [45] performed the best; however, superior results were achieved by combining the outputs of the three assemblers (see Supplementary Fig. 1). EvidentialGene was chosen as the preferred method of combining the assemblies, compared to the simple concatenation of the three assemblies and performing duplicate removal. We then checked whether there was a threshold of transcription for successful *pir* assembly by comparing the TPM expression of each gene to the quality of the best transcripts identified for the assemblies (see Supplementary Fig. 2). This demonstrated that even a small amount of transcription is enough for a *pir* to be constructed by the program, although quality is enhanced the more a gene is expressed, as expected. Only a few *pir* genes are both transcribed and not assembled to any degree, and these still are only found to be expressed at no higher than 100 TPM. Since EvidentialGene accomplished similar quality levels to simple assembly concatenation/de-duplication, but returned a smaller total number of transcripts, we suggest that it would be the better method to use for identifying unknown *pir*s with higher accuracy.

### De novo assembly of *Plasmodium vivax pir* genes

**Fig. 2:**
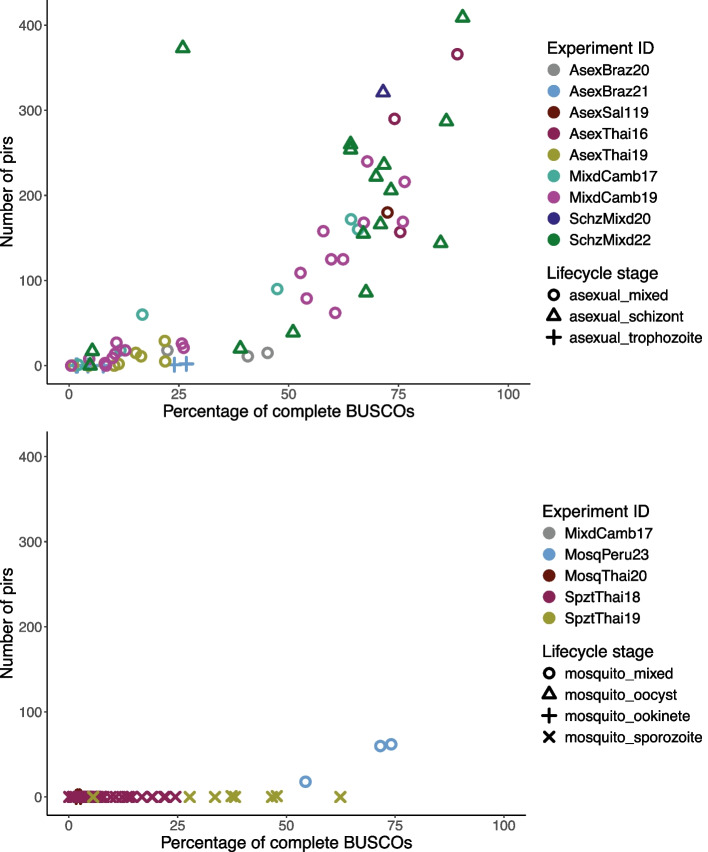
The number of *P. vivax pir*s found in each assembly compared to the percentage of complete BUSCOs (including duplicated). Colour of the points denotes the experimental origin and shape denotes the stage of the lifecycle that the parasites were isolated from. The top graph corresponds to the assemblies from blood samples containing asexual stages, and the bottom graph to those from infected mosquito samples. Full length *pir* transcripts were identified by covering ≧ 75% of the *Plasmodium*_*vir* Markov model (Pfam: PF05795) and were verified by being transcribed at ≧ 1 TPM in at least one sample used to produce that assembly

Fourteen transcriptome datasets were available from *P. vivax* samples, from multiple geographical sources and life-cycle stages (Table 1). The de novo transcriptomes of *P. vivax* covered a range of BUSCO quality scores and numbers of *pir* genes. Using the assemblies from *P.vivax* blood stages, which were generally of high BUSCO quality (> = 50%), we identified transcription of up to 400 *pir* genes per isolate (See Fig. 2). It was notable that the number of *pir*s detected does not plateau, even in the highest quality assemblies, so these assemblies were not reaching the total number of *pir*s expressed in these isolates.

Some assemblies, particularly from RNA of liver-stage and many mosquito-stage samples, showed both poor BUSCO quality and a low total number of *pir* mRNA transcripts, highlighting the difficulty of obtaining enough parasite RNA for next-generation sequencing from samples containing only a small proportion of parasite material (see Fig. 2A). However, there were assemblies from multiple sporozoite samples with acceptable BUSCO quality but still with no *pir* genes detected, suggesting that *pir* genes were not transcribed in sporozoites (see Fig. 2B). The only mosquito stage samples which showed evidence of *pir* expression were from the blood-meal samples of ‘MosqPeru23’, which is discussed in more detail at the end of the results.

From the asexual stages of *P. vivax* hundreds of *pir* transcripts have been successfully identified, demonstrating the feasibility of the de novo method to extract *pir* transcripts from the RNAseq reads themselves.

### demonstrating the feasibility of the *de novo* method to extract *pir* transcripts from the RNAseq reads themselves

The sub-families of the *Plasmodium vivax pir* genes were first defined by [8], and substantially updated by [7]. These sub-families have since been found throughout the *pir* repertoires of the *P. vivax* reference genomes P01, and W1 [19, 20], as well as other *P. vivax* genome assemblies and even some overlap with the *pir*s of other species [16]. We sought to determine whether these sub-families describe all of the sequence diversity present in the de novo* P. vivax pir* transcripts. To this end a BLAST similarity network [46] was constructed using the de novo sequences, the *pir*s of the Sal1, P01 and W1 *P. vivax* reference genomes, and the *pir*s of the reference genomes for the closely related species *P. knowlesi*, *P. vivax-like*, *P. ovale, P. malariae, P. cynomolgi, P. coatneyi*, and *P. brasilianum* [16, 47–52]. A highly interconnected network was produced; however, clusters could still be resolved (see Fig. 4).

**Fig. 3:**
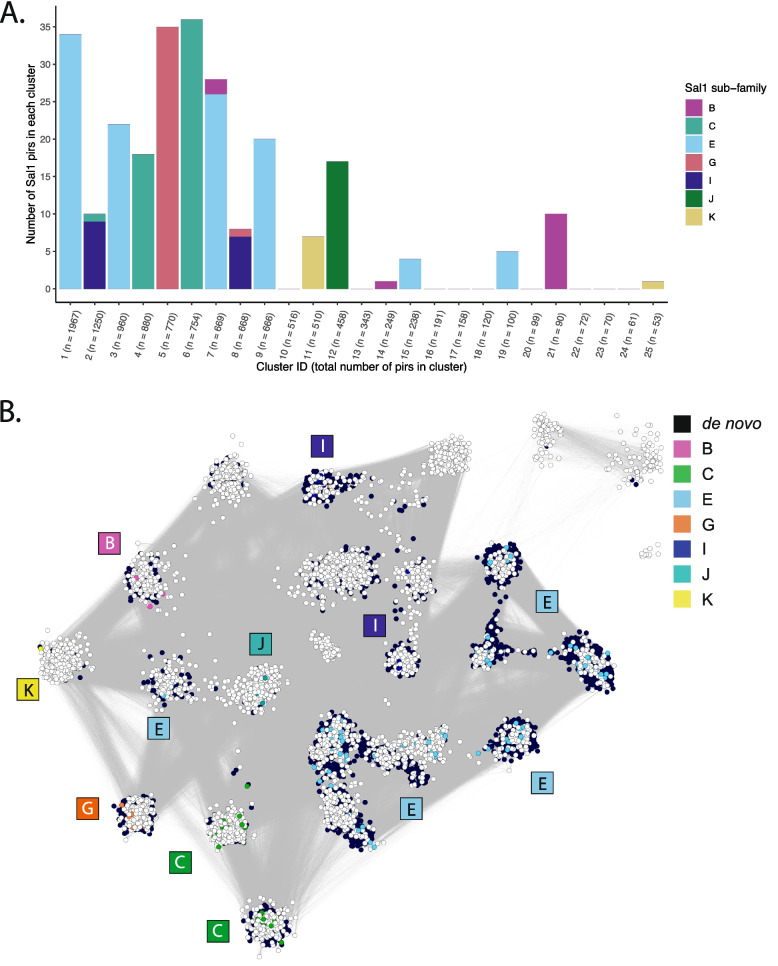
A. The number of *P. vivax* Sal1 sub-family member *pir*s in each cluster of the network, sorted by size. Cluster ID is named from the largest (Cluster 1) to the smallest (in this graph, Cluster 25). **B**. The *pir* sequence similarity network, with the Sal1 reference sub-family members and the de novo* pir*s coloured as shown in the legend in the figure. White nodes include the other *P. vivax* reference *pir*s (from P01 and W1) and other species

**Fig. 4:**
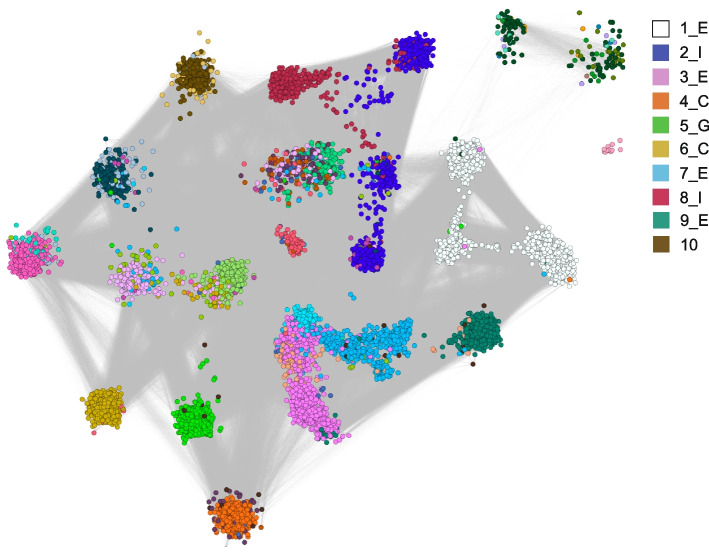
The BLAST similarity network of *pir*s, where the nodes are coloured by MCL clustering (with the largest ten clusters annotated in the legend

The clustering algorithm MCL [53] was used to identify sub-family clusters in the *pir* gene similarity network (consisting of reference genome, de novo, and non-*vivax pir*s), and these were compared to the previously defined sub-families from *P. vivax* Sal1. Overall, the existing sub-family designations aligned well with the MCL clustering (see Fig. 3). However, some clusters were not ascribed to any existing sub-families (e.g., cluster 10), and other existing sub-families were split between different groups (for example, sub-family E was split between clusters 1, 3, 7, 9, 15 and 19). Hence, an updated nomenclature was required. We propose simply naming the groups in order of size, appending the Sal1 name where members of the old sub-family were part of the cluster (new names shown as part of the BLAST network in Fig. 4). As an example, the largest cluster, which has many E sub-family sequences from the Sal1 genome, was termed 1_E by this method. The fourth largest cluster, which overlaps with C sequences from Sal1, was called 4_C. This 4_C grouping contains the highly conserved ancestral *pir* sequence, which has orthologs across all species with canonical *pir* members in their genomes and tends to be particularly highly transcribed. The largest cluster without any assigned Sal1 sequence (although it did contain unassigned Sal1 sequences) was cluster 10, demonstrating further how well the old definitions cover newer assemblies of *pir*s.

**Fig. 5:**
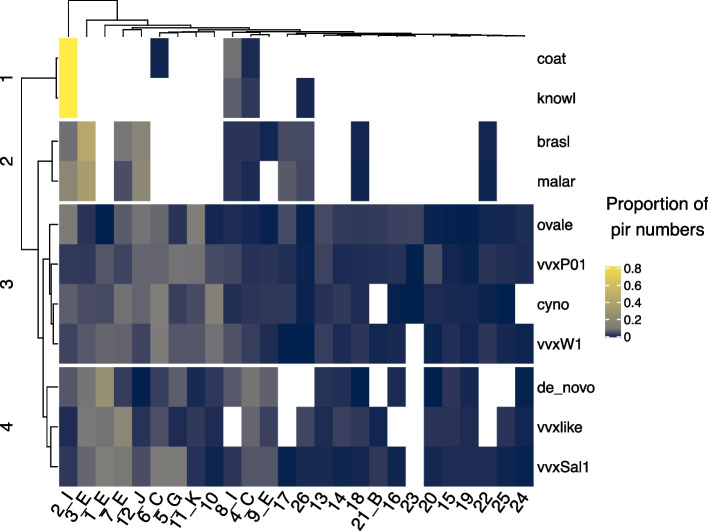
The proportion of numbers of *pir*s from each sub-family among the different genomes and this study’s de novo transcriptome. Each axis is ordered according to hierarchical clustering. The species/strain names are abbreviated as follows: *P. coatneyi*, coat; *P. knowlesi*, knowl; *P. brasilianum*, brasl; *P. malariae*, malar; *P. ovale*, ovale; *P. vivax* P01, vvxP01; *P. cynomolgi*, cyno; *P. vivax* W1, vvxW1; *P. vivax *de novo* pir* transcripts from this study, de_novo; *P. vivax-like*, vvxlike; *P. vivax* Sal1, vvxSal1. Note that the TPM colour scheme is skewed to lower values to prevent a few higher values dominating the heatmaps

The *P. vivax* sub-families show overlap with the *pir* repertoires of other species (see Supplementary Fig. 3), as previously observed [7, 15]. The smallest amount of cross-species sub-family sharing was with *P. coatneyi* and *P. knowlesi*, with only the 2_I (the largest sub-family in both *P. coatneyi* and *P. knowlesi*), 4_C (including the ancestral sequence), and 8_I families being shared between them and *P. vivax* (see Fig. 5). More *P. vivax* sub-families were shared with *P. malariae* and *P. brasilianum*, however almost all sub-families were shared between *P. vivax*, *P. cynomolgi, P. ovale,* and *P. vivax-like*. The currently available *P. vivax-like* genome, in contrast to every other species included, does not have representatives of group 8_I. Notably the sub-families 21_B, 17, 22, 23, and 25 were found in most of the *P. vivax* reference genomes but not the de novo* pir*s. It is unlikely that the pipeline is failing to capture these sub-families from the expression data, as running simulated RNAseq data of the reference genome *pir*s through the software did not suggest that these sub-families were systematically missed (see Supplementary Fig. 4). This supports the assertion that these groups were expressed at low levels (if at all) and would not be found in RNAseq data.

Using the larger repertoire of *pir* sequences obtained by de novo assembly we have refined the sub-family definitions for *pir*s from *P. vivax* and related species. Overall, the clusters match the previously defined reference sub-families with minor changes.

### Transcription of the de novo* pir* genes across geographies

**Fig. 6:**
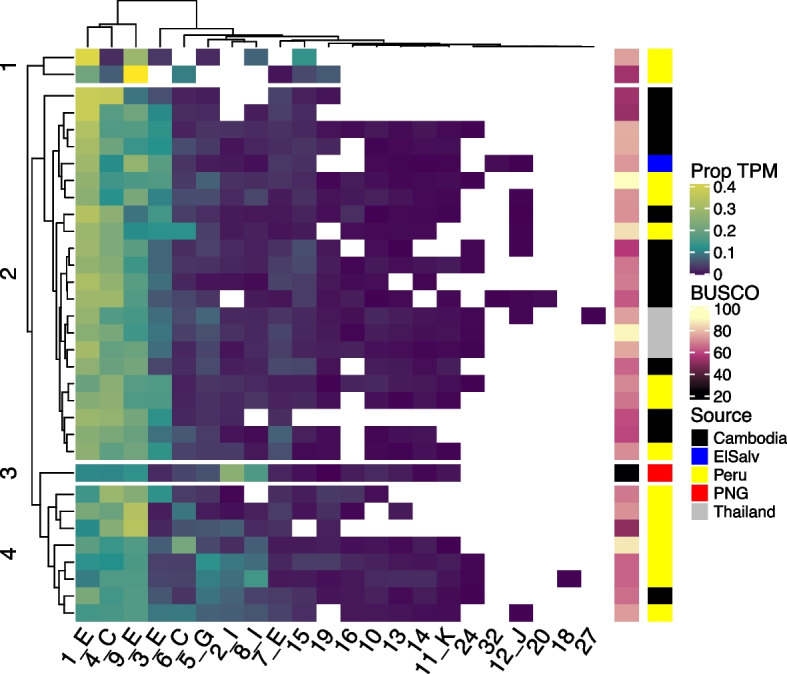
The expression of sub-families shown as the proportion of *pir* TPM from each sub-family among the samples of each assembly, alongside the corresponding BUSCO scores and geographic source. Each axis is ordered according to hierarchical clustering. Note that the TPM colour scheme is skewed to lower values to show the distinctions at smaller proportions. This figure only shows samples with > 50% BUSCO score, since many sub-families are missing from lower quality assemblies, although the SchzMixd22_Sch16 isolate (Schizont sample 16, from Papua New Guinea) was still included as it had a high *pir* number despite a relatively low BUSCO score

The new sub-family definitions were used to evaluate whether groups of *P. vivax pir* genes were differently transcribed across isolates from distant geographical locations. For this the RNAseq quantification (alignment and feature counting of the original reads to the de novo assembly) was compared. Broadly speaking, the alignment and quantification of the de novo* pir* transcription matched the results from the original studies, such as the pattern of transcription across the asexual cycle in Zhu et al., 2016 [28] (AsexThai16—see Supplementary Fig. 6), and the relative similarity of transcription between parasites obtained from two different species of splenectomised hosts in Gunalan et al., 2019 [34] (AsexSal119—see Supplementary Fig. 7). In Fig. 6 we show the gene expression (TPM proportions) of the *pir* sub-families in each isolate (for samples with higher *pir* numbers, for the sake of visualisation), and how this relates to BUSCO score and geographical origin. Most *pir* sub-families were present across these samples, and the larger groups always have representatives in the de novo transcriptomes. At this level the main distinguisher between the samples was the number of *pir* sub-families present, as many with the lower BUSCO scores have *pir* sub-family gaps. The proportion of *pir* numbers demonstrates a similar pattern (see Supplementary Fig. 5). Overall, the largest sub-family, 1_E, tends to dominate *P. vivax* expression profiles, followed by 4_C (the sub-family that includes the ancestral *pir* gene) and 9_E. The largest sub-family not defined in the reference Sal1 strain (‘new’ sub-families), cluster 10, did not appear to be transcribed very much at all. Other newly defined sub-families, 15 and 19, were transcribed more highly throughout the datasets, but still at a low level compared to the most highly expressed clusters.

There were few clear associations between *pir* sub-families and geographical locations. Hierarchical clustering separated most of the Peruvian transcriptomes (yellow rows in Fig. 6), into a distinct group, with a higher proportion of the subfamilies 5_G, 2_I, and 8_I. However, this was not fully consistent across the Peruvian samples, and these sub-families were highly expressed in some Cambodian samples. Indeed, a negative binomial model, considering BUSCO score, sub-family membership, geographic origin, and stages of the life cycle, demonstrated that there was no statistically significant linear relationship between Peruvian origin and any *pir* sub-family. The only geographical associations that arose from these statistical tests (adjusted *p* < 0.05) were for sub-families transcribed at low levels, and the effect size was so small that the relevance was questionable. Hence, we concluded that transcription of sub-families was consistent across *P. vivax* isolates from disparate geographical regions.

### De novo* pir* assemblies suggest *pir* transcription at the ookinete stage

**Fig. 7:**
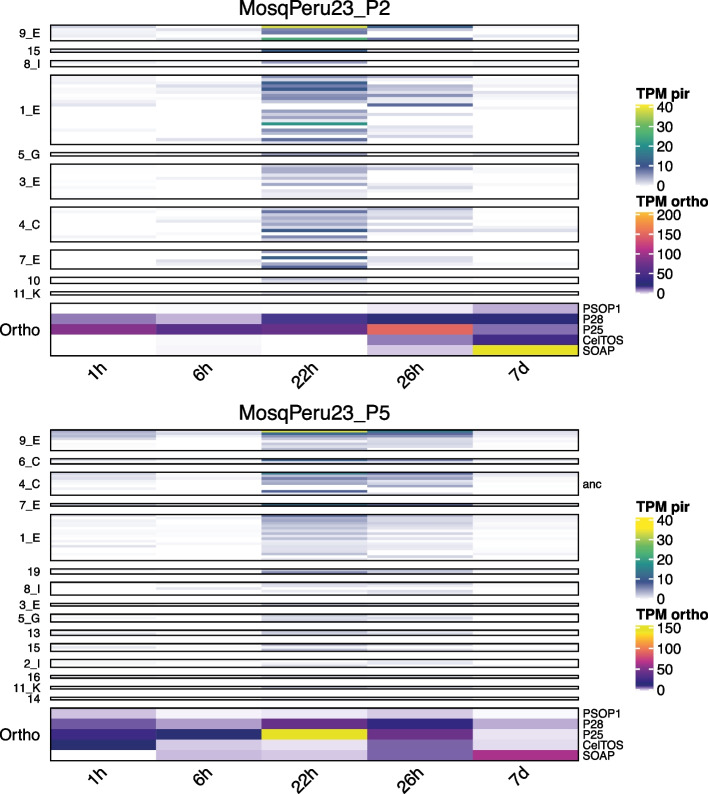
The expression (‘TPM pir’) of de novo assembled *pir* transcripts separated by sub-family across the timepoints (time after mosquito bloodmeal) of patient isolates from MosqPeru23. Right-hand label shows whether a *pir* transcript has >  = 99% identity to the ancestral *pir* gene (in this case only one *pir* transcript—from patient isolate P5 -assembled matched the ancestral copy to this degree). The heatmap box at the bottom of each heatmap, labelled ‘Ortho’, shows the transcription of selected orthologue transcripts that were the best matches of the conserved BUSCO orthologue, as named on the right-hand side of the heatmap body. The TPM colour scheme is separate for this heatmap and is labelled ‘TPM Ortho’. The gene names have been shortened as follows: PSOP1 (putative secreted ookinete protein 1); P25 and P28 (25 kDa and 28 kDa ookinete surface proteins); CelTOS (Cell-traversal protein for ookinetes and sporozoites); SOAP (secreted ookinete adhesive protein)

The analysis of most mosquito samples indicated that there were few or no *pir* genes transcribed in this part of the parasite lifecycle. The few mosquito stages from which we assembled *pir* transcripts (the MosqPeru23 samples) were taken from mosquito midgut bloodmeals at different times after feeding on infected Peruvian patients. From this experiment we included three different isolates (originating from three individual patients) and these three de novo assemblies all had > 50% Complete BUSCOs and included > 20 predicted *pir*s. We investigated these samples in more detail by analysing how *pir* TPM changed across the time-course of the experiment. Given that the mosquito bloodmeal would initially include surviving asexual stages, the presence of *pir* transcripts could be explained as a holdover from these cells instead of the parasite’s specific mosquito stages (beginning with gametes). If the asexual stages were the explanation for this signal, then we would expect the gene expression to dominate earlier post-feed and then diminish over time. Instead, *pir* transcription was minimal during the initial time points, but dominated at 22 and 26 h post-bloodmeal, although signal had vanished by 7 days (Fig. 7). This was compatible with the time of ookinete development of *P. vivax* [54], providing evidence for the presence of *pir* transcripts in the mosquito stages of this species. Few of these mosquito-specific *pir*s had 99% BLAST matches to any reference genome *pir*s, hence mapping to the reference genomes may have missed these signals. Overall, the TPM expression was reasonably low (< 40 TPM) but the peak was consistent across each of the three separate isolates (see Fig. 7 and Supplementary Fig. 8). An alternative explanation for this observed timing of *pir* transcription could be that the later time points simply have a higher number of total reads, with the only *pir*s of the overall assembly being extracted from these reads and missed from lower-read samples. The total counts of reads aligned to the de novo transcriptomes, however, were not necessarily higher in the 22/26 h time points compared to others (indeed, for isolate P2 the total number of aligned reads was lower for 22 h than it was at the 1 h or 6 h time points), so this is unlikely to be the explanation. The highest expression was concentrated within 9_E *pir*s, so future research could be conducted to ascertain whether there is a role of this sub-family in parasite-mosquito interactions.

## Discussion

The natural diversity of *P. vivax* and its *pir* multigene family make these loci particularly difficult to study. Gene expression studies, for example, are frustrated by the reference genomes for *P. vivax* only possessing some of the same *pir* sequences as wild isolates. To circumvent this problem and allow the study of *pir* gene expression from a diverse collection of *P.vivax* isolates, we employed de novo transcriptome assemblers to find the *pir* sequences from the RNAseq data themselves. Because of the sequence diversity between different isolates, we considered the best approach of analysis to be comparing transcription of sub-families instead of individual *pir* genes. Many of the assembled *pir*s are not shared between isolates, however the sub-families are a commonality, so this approach permitted us to analyse differential expression across the life cycle stages and geography.

A bespoke Nextflow pipeline [55] which employed the Trinity [56] and SPADES assemblers [45, 57], and combined them using EvidentialGene [42, 43], was successful in generating *P. c. chabaudi pir* transcripts, and therefore utilized in this study. Based on the reference genomes, we would expect an upper ceiling of ~ 1000 *pir*s from the assembly of each isolate, although the highest number of recoverable transcripts will be lower as some of these *pir*s will not have enough coverage in the read data. With our pipeline, we found up to ~ 400 *pir* transcripts expressed by any one isolate, thousands in total across the data, covering a range of life cycle stages, geographical sources, and *pir* sub-families. From sequence similarity networks of these de novo transcripts, we refined the *pir* sub-family definitions and demonstrated that they are generally transcribed in similar patterns in isolates worldwide. Gene expression of the *pir*s across the life cycle re-affirmed a burst of transcription in the mosquito stages of development previously seen in *P. berghei*, suggesting a role for this gene family in the vector of human-infecting parasites.

Using the BUSCO [44] score to quantify the number of *Plasmodium sp.*-conserved transcripts in each assembly (as a metric of overall quality), it was clear that the numbers of *pir* transcripts being produced was related strongly to the completeness of the overall de novo transcriptome. The numbers of transcribed *pir*s showed no evidence of plateauing even with high-quality assemblies, so it is likely that the total *P. vivax* expressed *pir* repertoire was underestimated. Hundreds of transcribed *pir*s may not be assembled by this method either due to limitations of the data and tools, and/or because the missing *pir*s themselves were transcribed at too low a level to be picked up. *Pir*s could be missed by the hidden Markov model or be excluded by the model coverage threshold. The number of *pir* sub-families found to be expressed in each assembly is also dependent on the sample quality and sequencing depth, meaning that some families may be absent due to the lack of detected reads even if they are biologically still present. This presents a limitation for concluding whether sub-families are present in given life-cycle stages or geographical regions.

Comparison of the transcriptome assembly tools threw up some further surprises. Even though rnaSPAdes was developed specifically for transcriptomes, its sister implementation SPAdes (using the option developed for single-cell genome assembly) gave similar results. A comparison by Holzer & Marz, 2019, between many assemblers, observed the comparable and sometimes even superior performance of SPAdes (single-cell) across multiple metrics, so the single-cell specific algorithm does improve transcript assembly in certain contexts. Trinity is often the default choice for bioinformaticians, including in some of the original studies included in this analysis [27, 28], however it was much less effective for the assembling of *pir* genes than the SPAdes tools. Trinity also produced the lowest BUSCO scores, however the decline in assembly quality was small, while the decline in *pir* transcript recovery was stark. Since *pir*s are generally transcribed at low levels, the scant reads from these transcripts may have been removed by Trinity’s initial k-mer ranking and filtering algorithm. A future execution of this workflow could include more de novo transcriptome assemblers to potentially expand and improve upon the repertoire of *pir*s detected. Each assembler has its own strengths and weaknesses and can work better for certain species over others [58–65], so one stands to gain further transcriptional insight by including more tools like Bridger [66], Trans-ABySS [67], and SOAPdenovo-trans [68], among others. EvidentialGene worked well to create a meta-assembly from the outputs of the three individual assemblers, proving the usefulness of its algorithms in such a pipeline. This software is not as fully documented as many other pieces of bioinformatic programming, and it would benefit from more expansive explanations.

Much of our understanding of putative *pir* functions and transcriptional kinetics comes from rodent-infecting parasites. Consistent evidence from *P. berghei,* across multiple stages of the life cycle and different experiments, show some expression in the mosquito (especially at the oocyst stage) and increasing expression upon entering the mammalian host [18, 41, 69, 70]. Here we found—from one experiment, MosqPeru23—that around 22 h after *P. vivax*-infected bloodmeal uptake by the mosquito there is a small but consistent surge in *pir* transcripts, coinciding with the conversion of zygotes to ookinetes. A signal from the 9_E sub-family was notably strong at this time-point, suggesting that its members have a role in the mosquito. To our knowledge this is the first indication that ookinetes of a human-infective *Plasmodium spp.* express *pir* genes, following on from the first mosquito-stage *pir* transcription identified in the *P. berghei* malaria cell atlas [70]. Together these data suggest that some *pir* genes may have a role in the mosquito vector.

We were not able to gain insight into the *pir* transcriptomes of certain life-cycle stages, such as liver stages due to a lack of appropriate data. Gural et al., 2018 [29], had to contend with a massive abundance of host mRNA compared to parasite mRNA, and so they employed hybrid capture sequences to enrich for the *P. vivax* transcripts. The capture sequences were based on the P01 reference genome, so it is unlikely that the sequencing results would have contained novel *pir* sequences for extraction. For the murine-infective parasite *P. berghei* the malaria cell atlas [70] has shown trophozoite-like *pir* transcription in the liver stages, and [69], have shown that PIR proteins are expressed in the late liver stages, demonstrating that the genes may play a role also in the exo-erythrocytic stages of other malaria parasites and represent an important avenue of future work.

*Pir* genes of both *P. berghei* and *P. chabaudi* demonstrate especially high transcription during the asexual blood stages, however *P. berghei* shows greater sexual dimorphism of *pir* transcription (with male gametocytes enriched for *pir* expression) [71]. *P. chabaudi* infections in mice particularly demonstrate the importance of the gene family in the rodent asexual stages, providing evidence of an association between *pir* transcription and the virulence of infection or chronic recrudescence [11, 12]. We can make comparisons between rodent *pir* transcription and the *P. vivax* life-cycle stages that gave good quality assemblies. The data of [28] (AsexThai16), which sampled across the intra-eyrthrocytic development cycle (IDC) of *P. vivax*, suggest that although *pir* transcription is unimodal over the cycle, the timing of this peak within the asexual cycle is different for *P. vivax* and *P. c. chabaudi* [41]; a result observable in the original paper and verified here (Supplementary Fig. 6). For *P. c. chabaudi* the peak of *pir* transcription occurs around the time of schizont differentiation, while for *P. vivax* the peak is around schizont bursting and ring-stage formation. Microarray comparisons between multiple *Plasmodium sp.* using 1–1 orthologs found that the genes with the most variation in timing across the genus were enriched for those transcribed primarily during the early ring and early schizont stages, so it may be common for IDC genes to show altered patterns of expression [72]. The interpretation of the *P. vivax* IDC data is complicated by the fact that it is generated from in vitro culture, especially problematic at the 48 h time-points when toxicity of the culture could be causing artefacts (*P. vivax* cannot be cultured long-term [73–75]). Investigations from natural infections are impeded by the asynchronous nature of the *P. vivax* IDC. Single-cell RNAseq provides the best opportunity to gain access to the individual stages of the IDC. Additionally, sequencing of sexual stages would be of additional interest since the mouse-infective species *P. berghei* and *P. c. chabaudi* show contrasting patterns of *pir* transcription between sexes; both show enriched expression in males, although only *P. c. chabaudi* has a female gametocyte-specific subset too [71]. Although there is a conserved transcription of *pir*s across malaria species, the exact timing may differ between simian- and mice-infective clades.

We used a BLAST similarity network to define *P. vivax* sub-families, showing that the groupings previously annotated before high-throughput sequencing broadly still describe the community structure of gene sequences found in isolates obtained worldwide. This was already suggested by existing reference genome assemblies, which originated from different countries but all had overlapping sequences. Nonetheless, our similarity network suggested that some definitions needed updating, such as the largest sub-family E which is better described as multiple sub-families. Clusters on the network (corresponding to sub-families) of note include the largest sub-family 1_E, which tends to dominate *P. vivax* expression profiles, and 4_C, the sub-family that includes the ancestral *pir* gene for each isolate [21]. It is curious that the ancestral *pir* is part of a greater sub-family instead of on a lone lineage, as is observed in phylogenies of the murine-infective *Plasmodium sp. pir*s. Similar to other species the ancestral *pir* is often highly transcribed, however many de novo assemblies did not include the sequence. This could be because the assembly software did not ‘find’ this transcript among the RNAseq reads. Only two of the *P. c. chabaudi *de novo assemblies did not give rise to > 95% identity BLAST matches of the ancestral *pir*, and these were both from the outputs of individual assembler tools (all the combined assemblies assembled accurate full-length ancestral transcripts). Transcription of the ancestral *pir* may be relatively lower in *P. vivax* compared to other species, including *P. c. chabaudi* [41]. If the ancestral *pir* is downregulated in *P. vivax* compared to the rodent malaria parasites, this could be due to functional redundancy from the other similar members of the 4_C *vivax pir* sub-family. Other pre-defined sub-families showed persistently low transcription/presence in the assemblies, including cluster 11 (K), cluster 12 (J), and cluster 21 (B). These *pir*s are likely un-expressed and may only be found through genome sequencing.

Since this study used RNAseq data, we tested whether the distribution of *pir* sub-families across geographical localities held true for *pir*s that are actually transcribed, and indeed it did. Even when statistical tests were conducted to test for differences in *pir* sub-family transcription across the world, only small associations of questionable relevance were found. Evidence suggests that, with a few exceptions, the genotypes of wild *P. vivax* isolates tend to cluster by their geographic source [76]. A particularly recent founder effect can be found in the American populations of *P. vivax*, which likely derive from European colonization [76, 77]. There does not appear to be any reflection of these geographically restricted clades in the data presented here. If we assume that the spread of *P. vivax* strains through human movement has not overwritten regional distinctions, this result suggests that the sub-families of the *pir*s are relatively unchanged since the last common ancestor of *P. vivax*, and hence they either have a purpose that the parasite needs to preserve, or that not enough time has passed for them to significantly diverge. Estimates of the timing of the most recent common ancestor of *P. vivax* range from around 50–300,000 years ago [24, 78–80], a relatively short evolutionary time. However, the genetic variation of multigene families like the *pir*s should lead to accelerated change and loss/gain of sub-family copy numbers or transcriptional rates. Expression differences of individual *pir* genes between samples from different continents have been observed before when aligning to the P01 genome *pir*s [81], however we suggest that this does not expand to the overall sub-families themselves. New *P. vivax* genomes/transcriptomes from different regions of the world continue to be sequenced, offering opportunities to incorporate them into the pipeline and challenge this conclusion [81–83]. Some isolates of interest may be found from patients on the China-Myanmar border in the upper Mekong, which have previously been shown to have a particularly large C sub-family [84].Only a single Papua New Guinean sample was available for this study, however it showed a distinct profile and was an outlier in the relationship between BUSCO score and *pir* number (having a potent abundance of *pir*s despite a relatively incomplete assembly). Analysis of orthologue diversity across *P. vivax* isolates from multiple countries demonstrated that PNG parasites were particularly diverse compared to those from other regions [76], so it is plausible that this variation is reflected in multigene families too. Deep RNA-sequencing and de novo assembly of PNG-sourced *P. vivax* transcriptomes could shed light on whether the *pir*s of these pathogens are unique to the country.

De novo transcriptomics can be employed across diverse *P. vivax* RNAseq datasets in order to observe the transcription of *pir* sequences missing from the reference genomes. This method unveiled a refined sub-family structure and demonstrated that these sub-families are expressed in dispersed parasite isolates around the world, suggesting that they have functions that lead to their retention. Although thought previously to have no role in mosquito stages, now evidence exists from both *P. berghei* and *P. vivax* that there is indeed *pir* gene expression in the vector, opening new opportunities to understand this gene family.

## Materials and methods

### Brief experimental information from Carlos et al., unpublished (MosqPeru23)

Female *An. darlingi* mosquitoes were fed on three patients (P2, P5, and P6) from Iquitos, Peru, with active *P. vivax* infections as determined from blood smears. At each timepoint post-bloodmeal and for each patient isolate 30–45 midguts were dissected and the RNA processed for Illumina NextSeq 500 sequencing.

### *Plasmodium spp.* data download

*P. vivax* datasets were downloaded using the FetchNGS pipeline v1.5 available from nf-core using Nextflow v22.10.3 and Singularity v3.6.4. Default settings were used except for ‘–force_sratools_download’ to use SRA-toolkit for the download, and an additional custom config file (https://github.com/timslittle/Thesis_Github_221023/blob/main/custom.config) supplied via ‘-c’ to permit the SRA-toolkit function ‘prefetch’ to use larger files than default. The configuration profile used for all Nextflow pipelines was ‘Singularity’ and a locally maintained Crick profile (https://github.com/nf-core/configs/blob/master/docs/crick.md). The exceptions were the Carlos et al. (manuscript in preparation) samples and the *P. c. chabaudi* test samples, which were downloaded directly from lab storage, and the [39] samples, which were downloaded from cloud storage (kindly provided by the authors).

At this point files were concatenated together if they constituted the same isolate of *P. vivax*, to maximise the amount of information for the transcriptome assemblers, but minimise the erroneous assembly of transcripts from stitching reads of different sources. Where data source was unclear from the original publication, the samples were kept separate for assembly, such as for [30]. See Supp File 1 for the full list of files concatenated together and the rationale for this.

### De novo transcriptome assembly pipeline with Nextflow

The pipeline for de novo assembly construction, ‘transcript_corral’ (development version 1, commit: 6b93401, https://github.com/timslittle/nf-core-transcriptcorral/tree/6b93401496098d2759e875a7611cf0eb1b4268c4) was written with Nextflow v22.10.3 and nf-core/tools v2.7.dev0. The pipeline was submitted with the parameters: ‘-profile singularity,crick –skip_trimming false –remove_ribo_rna true –filter_genome [/path/to/genome_to_filter.fa] –assemble_trinity true –assemble_spades_sc true –assemble_spades_rna true –use_evigene true –hmmsearch_hmmfile [/path/to/Pfam-Plasmodium_Vir.hmm] –busco_lineage'plasmodium_odb10'–salmon_alignment true –salmon_gtf false’. In summary, the pipeline begins by concatenating files that require concatenation (see above), running Trim_Galore! v0.6.7 (which uses cutadapt v3.4) to trim adapter sequences, and running FastQC (v0.11.9) for quality analysis. Reads within the RNAseq datasets that align to potential contaminant genomes were then removed using HISAT2 v2.1.0 with parameters ‘-q -x –un-conc-gz’, with the last parameter saving the sequences which do not align concordantly to the reference [85]. The contaminant genome was a concatenation of the *Mus musculus* genome (due to the mouse fibroblasts in the [29] system), the *Homo sapiens* genome GRCh38 [86], the mosquito *Anopheles dirus* WRAIR2 AdirW1 genome (due to the mosquito-sourced samples of [36]) [87, 88] and *Anopheles darlingii* AdarC3 (due to the Carlos et al., unpublished, samples) [89], as well as the primate *Saimiri boliviensis* SaiBol1 genome [90] and *Aotus nancymaae* GCA_000952055.2 genome [91] (the monkeys used in [34]). The HISAT2 alignment removal was performed with the relevant parameters altered for the single-end and paired-end stranded *P. vivax* samples: for the stranded libraries of Kim et al., 2017 and 2019 [27, 33] samples HISAT2 was performed with ‘–rna-strandness FR’; for the single-end libraries of Muller et al., 2019 [32], and Gural et al., 2018 [29], HISAT2 was performed with ‘–un-gz’ instead of ‘–un-conc-gz’. rRNA reads were subsequently removed using sortmerna v4.3.4 [92]. These output sequences were then used for assembly using sc-spades and rna-spades (SPAdes v3.15.4 with options ‘–sc’ and ‘–rna’ respectively), and Trinity v2.13.2. The assemblies were combined, and redundant sequences removed, using EvidentialGene version 22 may07, which assesses similar sequences for the most optimal representative transcript/peptide [42, 43]. In brief, CDS sequences are identified and scored, perfect duplicates and fragments are removed, alternative transcripts identified using 98% identity BLAST alignments of coding sequence, and a primary transcript is finally identified. To ascertain the quality of the final meta-assemblies they were ran through BUSCO v5.4.3 with ‘plasmodium_odb10’ as the reference, to see how many expected, conserved *Plasmodium* genes are recovered [44, 93]. HMMer v3.3.2 is used to identify the sequences which resemble the Plasmodium_Vir Pfam model (PF05795) [94], then matches with an E-value of less than 1e-3 were extracted [95]. Transcriptional quantification of the samples was used to both detect which of the proposed *pir* transcripts have RNAseq reads map back to them, and to evaluate how the *pir*s are being transcribed. All the original biological replicates (after Trim_Galore!, HISAT2, and sortmerna processing) were aligned to their corresponding assemblies using the transcript-aware aligner Salmon v1.9.0 [96]. The parameters ‘-p 8 –validateMappings -I A’ were used for all alignments, with ‘-l SR’ specified instead of ‘-I A’ for the stranded libraries. Salmon calculates TPM automatically, so these results were used directly to filter the HMMer ORF outputs for the PIR sequences with evidence of transcription (greater than or equal to 1 TPM in at least one sample). The pipeline was run in separate batches to reduce load on computational resources at any given time.

To check whether the pipeline failed to assemble/identify any given sub-families of *pir* genes, simulated RNAseq reads were generated based on the Sal1, P01, and W1 reference genomes (excluding duplicate sequences) using Rsubread v 2.0.1 [97] simReads with ‘paired.end = TRUE’ and all relative TPMs set to 1 (ensuring that all transcripts are ‘expressed’ at the same level, controlling for transcript length). The simulated RNAseq fastq files were ran through the pipeline as described above, and the output *pir* transcripts were compared to the reference genome *pir*s using tBLASTn v2.9.0 (‘-evalue 1e-3 -max_target_seqs 500 -outfmt"6 std qcovs qcovhsp slen nident’).

For the mosquito blood-meal RNAseq results of MosqPeru23 (Carlos et al., manuscript in preparation), the 18 h samples were excluded from the final figure due to this timepoint consistently showing globally distinct transcriptional profiles with zero *pir* expression. The reason for this is unclear, and may be due to biological signal (e.g. a transition time for the parasite between zygote and ookinete forms) or a technical artefact.

### *P. c. chabaudi* AS benchmarking of the de novo transcriptome method to find *pir* genes

The 24 h asexual cycle samples from Little and Cunningham et al., 2021, [41] were used to test this method for finding *pir* genes in RNAseq datasets, The Nextflow pipeline was run for each assembler separately, then with all assemblers together to produce a meta-assembly. To remove duplicates from these assemblies they were processed by CD-HIT v 4.8.1. To compare the use of EvidentialGene for meta-assembly construction, the meta-assembly was also run through EvidentialGene instead of CD-HIT. Instead of running HMMer and finding *pir* sequence, the peptide outputs of the pipeline were matched to the known PIR peptides from PlasmoDB v61 using BLAST v2.9.0 with parameters “-evalue 1e-3 -num_threads 6 -max_target_seqs 500 -outfmt"6 std qcovs qcovhsp slen nident".

### Creating networks of PIRs and sub-family assignment

To create networks of the PIR ORFs and work out which sub-family they belong to/how well existing definitions describe these PIRs, I downloaded the already published PIR sequences from *P. vivax* strains P01, Sal1, and W1, and *P. ovale*, *P. vivax-like*, *P. malariae, P. brasilianum, P. knowlesi, P. coatneyi,* and *P. cynomolgi*, using a PlasmoDB v62 search for “PIR protein” or “VIR protein” in ‘Product Description’, as well as the a Pfam search for PF05795: Plasmodium_Vir and PF06022: Cir_Bir_Yir Variant antigen (adding the results of both the text and PFAM search together), then finally filtering out pseudogenes. This found 316 Sal1, 610 W1, and 1098 P01 *P. vivax pir*s. These ‘known’ PIRs were combined with the de novo PIRs and BLASTp v2.9.0 was performed between this dataset and itself (‘-evalue 1e-3 -num_threads 6 -max_target_seqs 500 -outfmt"6 std qcovs qcovhsp slen nident"’).

MCL clustering was performed with varying inflation values (influencing the number of clusters) of 1.2, 1.4, 1.8, 2, 2.5, 3, 4 and 6 (mcxload parameters: ‘–stream-mirror –stream-neg-log10 -stream-tf'ceil(200)'’; mcl and mcxdump parameters all default) [53]. As reported in the results, the best compromise between minimal total number of clusters and minimal mixing of Sal1 sub-family sequences was found with an MCL inflation value of 1.4. From these MCL clusters sub-families were assigned based on the majority Sal1 sub-family sequence in the cluster. If a cluster had no assigned Sal1 sub-family sequence (note that there may be unassigned Sal1 sequence present which were not identified as belonging to any sub-family in the original study) then it was marked as a ‘New’ sub-family. These were then numbered if the assigned name was not unique.

The network was visualised using Gephi [98] and the OpenOrd layout algorithm [99] with 25% Liquid stage, 100% Expansion stage, 15% Cooldown stage and no Crunch or Simmer stage of the simulated annealing process, 0.8 edge cut, 7 threads, 750 iterations, 0.2 fixed time and a random seed of −9, followed by briefly running the Noverlap algorithm to reduce overlapping nodes with speed set to 3, ratio 0.5 and margin 5.

### Statistical analysis and figure production in R

Data analysis was conducted using stringr [100], dplyr [101] and data.table [102], figures were drawn using ggplot2 [103], ComplexHeatmap [104], circlize [105] and viridis [106] packages in R v3.6.2 [107]. The negative binomial model was fitted using the base R function ‘glm.nb’ with the formula ‘TPM ~ sub-family * country * lifecycle_stage + BUSCO_score’.

## Supplementary Information

Additional file 1. 

Additional file 2

Additional file 3. 

Additional file 4

Additional file 5. Supplementary Figure 1. 

Additional file 6. Supplementary Figure 2

Additional file 7. Supplementary Figure 3

Additional file 8. Supplementary Figure 4

Additional file 9. Supplementary Figure 5

Additional file 10. Supplementary Figure 6

Additional file 11. Supplementary Figure 7

Additional file 12. Supplementary Figure 8

## Data Availability

Data is provided within the additional information files. Source sequences are available on public databases. ImpMosq23 (Carlos et al., in preparation) data is available at ENA (PRJEB29445). The Gephi graph file and assembly sequence files are obtainable at doi:10.5281/zenodo.15384231. [To editor: Zenodo link is embargoed until the end of May].

## Abbreviations

*Pir*s: *Plasmodium interspersed repeats*
HMM: Hidden Markov Model
RNAseq: High-throughput RNA sequencing
BUSCO: Benchmarking Universal Single-Copy Orthologs
TPM: Transcripts-per-Million
IDC: Intra-eyrthrocytic Development Cycle

## References

[1] WHO. World Malaria Report 2021. Geneva; 2021. https://www.who.int/teams/global-malaria-programme/reports/world-malaria-report-2021.

[2] Baird JK. African *Plasmodium vivax* malaria improbably rare or benign. Trends Parasitol. 2022;38:683. 35667992 10.1016/j.pt.2022.05.006

[3] Culleton R, Ndounga M, Zeyrek FY, Coban C, Casimiro PN, Takeo S, et al. Evidence for the transmission of *Plasmodium vivax* in the Republic of the Congo. West Central Africa J Infect Dis. 2009;200:1465–9. 19803728 10.1086/644510

[4] Kho S, Qotrunnada L, Leonardo L, Andries B, Wardani PAI, Fricot A, et al. Hidden biomass of intact malaria parasites in the human spleen. N Engl J Med. 2021;384:2067–9. 34042394 10.1056/NEJMc2023884

[5] Reid AJ. Large, rapidly evolving gene families are at the forefront of host–parasite interactions in Apicomplexa. Parasitology. 2015;142:S57-70. 25257746 10.1017/S0031182014001528 PMC4413850

[6] Janssen CS, Phillips RS, Turner MR, Barret MP. *Plasmodium interspersed repeats*: The major multigene superfamily of malaria parasites. Nucleic Acids Res. 2004;32:5712–20. 15507685 10.1093/nar/gkh907 PMC528792

[7] Lopez FJ, Bernabeu M, Fernandez-Becerra C, del Portillo HA. A new computational approach redefines the subtelomeric *vir* superfamily of *Plasmodium vivax*. BMC Genomics. 2013;14:8. 23324551 10.1186/1471-2164-14-8 PMC3566924

[8] del Portillo HA, Fernandez-Becerra C, Bowman S, Oliver K, Preuss M, Sanchez CP, et al. A superfamily of variant genes encoded in the subtelomeric region of *Plasmodium vivax*. Nature. 2001;410:839–42. 11298455 10.1038/35071118

[9] Su X, Heatwole VM, Wertheimer SP, Guinet F, Herrfeldt JA, Peterson DS, et al. The large diverse gene family *var* encodes proteins involved in cytoadherence and antigenic variation of *Plasmodium falciparum*-infected erythrocytes. Cell. 1995;82:89–100. 7606788 10.1016/0092-8674(95)90055-1

[10] Fried M, Duffy PE. Adherence of *Plasmodium falciparum* to Chondroitin sulfate a in the human placenta. Science. 1979;1996(272):1502–4. 10.1126/science.272.5267.1502 8633247

[11] Spence PJ, Jarra W, Lévy P, Reid AJ, Chappell L, Brugat T, et al. Vector transmission regulates immune control of *Plasmodium* virulence. Nature. 2013;498:228–31. 23719378 10.1038/nature12231 PMC3784817

[12] Brugat T, Reid AJ, Lin J, Cunningham D, Tumwine I, Kushinga G, et al. Antibody-independent mechanisms regulate the establishment of chronic *Plasmodium* infection. Nat Microbiol. 2017;2:16276. 28165471 10.1038/nmicrobiol.2016.276 PMC5373435

[13] Bernabeu M, Lopez FJ, Ferrer M, Martin-Jaular L, Razaname A, Corradin G, et al. Functional analysis of *Plasmodium vivax* VIR proteins reveals different subcellular localizations and cytoadherence to the ICAM-1 endothelial receptor. Cell Microbiol. 2012;14:386–400. 22103402 10.1111/j.1462-5822.2011.01726.x

[14] Fernandez-Becerra C, Bernabeu M, Castellanos A, Correa BR, Obadia T, Ramirez M, et al. *Plasmodium vivax* spleen-dependent genes encode antigens associated with cytoadhesion and clinical protection. Proceedings of the National Academy of Sciences. 2020;117(23):13056-13065:201920596. 10.1073/pnas.1920596117 PMC7293605 32439708

[15] Ansari HR, Templeton TJ, Subudhi AK, Ramaprasad A, Tang J, Lu F, et al. Genome-scale comparison of expanded gene families in *Plasmodium ovale wallikeri* and *Plasmodium ovale curtisi* with *Plasmodium malariae* and with other Plasmodium species. Int J Parasitol. 2016;46:685–96. 27392654 10.1016/j.ijpara.2016.05.009

[16] Rutledge GG, Böhme U, Sanders M, Reid AJ, Cotton JA, Maiga-Ascofare O, et al. *Plasmodium malariae* and *P. ovale* genomes provide insights into malaria parasite evolution. Nature. 2017;542:101–4. 28117441 10.1038/nature21038 PMC5326575

[17] Carlton JM, Angiuoli SV, Suh BB, Kooij TW, Pertea M, Silva JC, et al. Genome sequence and comparative analysis of the model rodent malaria parasite *Plasmodium yoelii yoelii*. Nature. 2002;419:512–9. 12368865 10.1038/nature01099

[18] Otto TD, Böhme U, Jackson AP, Hunt M, Franke-Fayard B, Hoeijmakers WAM, et al. A comprehensive evaluation of rodent malaria parasite genomes and gene expression. BMC Biol. 2014;12:86. 25359557 10.1186/s12915-014-0086-0 PMC4242472

[19] Auburn S, Böhme U, Steinbiss S, Trimarsanto H, Hostetler J, Sanders M, et al. A new *Plasmodium vivax* reference sequence with improved assembly of the subtelomeres reveals an abundance of *pir* genes. Wellcome Open Res. 2016;1:4. 28008421 10.12688/wellcomeopenres.9876.1 PMC5172418

[20] Minassian AM, Themistocleous Y, Silk SE, Barrett JR, Kemp A, Quinkert D, et al. Controlled human malaria infection with a clone of *Plasmodium vivax* with high-quality genome assembly. JCI Insight. 2021;6:e152465. 34609964 10.1172/jci.insight.152465 PMC8675201

[21] Frech C, Chen N. Variant surface antigens of malaria parasites: functional and evolutionary insights from comparative gene family classification and analysis. BMC Genomics. 2013;14:427. 23805789 10.1186/1471-2164-14-427 PMC3747859

[22] Merino EF, Fernandez-Becerra C, Durham AM, Ferreira JE, Tumilasci VF, d’Arc-Neves J, et al. Multi-character population study of the *vir* subtelomeric multigene superfamily of *Plasmodium vivax*, a major human malaria parasite. Mol Biochem Parasitol. 2006;149:10–6. 16730808 10.1016/j.molbiopara.2006.04.002

[23] Chen S-BB, Wang Y, Kassegne K, Xu B, Shen H-MM, Chen J-HH. Whole-genome sequencing of a *Plasmodium vivax* clinical isolate exhibits geographical characteristics and high genetic variation in China-Myanmar border area. BMC Genomics. 2017;18:131. 28166727 10.1186/s12864-017-3523-y PMC5294834

[24] Neafsey DE, Galinsky K, Jiang RHY, Young L, Sykes SM, Saif S, et al. The malaria parasite *Plasmodium vivax* exhibits greater genetic diversity than *Plasmodium falciparum*. Nat Genet. 2012;44:1046–50. 22863733 10.1038/ng.2373 PMC3432710

[25] Zhang C, Oguz C, Huse S, Xia L, Wu J, Peng YC, et al. Genome sequence, transcriptome, and annotation of rodent malaria parasite *Plasmodium yoelii nigeriensis* N67. BMC Genomics. 2021;22:1–12. 33902452 10.1186/s12864-021-07555-9 PMC8072299

[26] Brashear AM, Roobsoong W, Siddiqui FA, Nguitragool W, Sattabongkot J, López-Uribe MM, et al. A glance of the blood stage transcriptome of a Southeast Asian *Plasmodium ovale* isolate. PLoS Negl Trop Dis. 2019;13: e0007850. 31730621 10.1371/journal.pntd.0007850 PMC6881071

[27] Kim A, Popovici J, Vantaux A, Samreth R, Bin S, Kim S, et al. Characterization of *P. vivax* blood stage transcriptomes from field isolates reveals similarities among infections and complex gene isoforms. Sci Rep. 2017;7:7761. 28798400 10.1038/s41598-017-07275-9 PMC5552866

[28] Zhu L, Mok S, Imwong M, Jaidee A, Russell B, Nosten F, et al. New insights into the *Plasmodium vivax* transcriptome using RNA-Seq. Sci Rep. 2016;6:20498. 26858037 10.1038/srep20498 PMC4746618

[29] Gural N, Mancio-Silva L, Miller AB, Galstian A, Butty VL, Levine SS, et al. In vitro culture, drug sensitivity, and transcriptome of *Plasmodium vivax* hypnozoites. Cell Host Microbe. 2018;23:395-406.e4. 29478773 10.1016/j.chom.2018.01.002 PMC8048090

[30] Roth A, Adapa SR, Zhang M, Liao X, Saxena V, Goffe R, et al. Unraveling the *Plasmodium vivax* sporozoite transcriptional journey from mosquito vector to human host. Sci Rep. 2018;8:12183. 30111801 10.1038/s41598-018-30713-1 PMC6093925

[31] Cheng CW, Jongwutiwes S, Putaporntip C, Jackson AP. Clinical expression and antigenic profiles of a Plasmodium vivax vaccine candidate: merozoite surface protein 7 (PvMSP-7). Malar J. 2019;18:197. 31196098 10.1186/s12936-019-2826-7 PMC6567670

[32] Muller I, Jex AR, Kappe SHI, Mikolajczak SA, Sattabongkot J, Patrapuvich R, et al. Transcriptome and histone epigenome of *Plasmodium vivax* salivary-gland sporozoites point to tight regulatory control and mechanisms for liver-stage differentiation in relapsing malaria. Int J Parasitol. 2019;49:501–13. 31071319 10.1016/j.ijpara.2019.02.007 PMC9973533

[33] Kim A, Popovici J, Menard D, Serre D. *Plasmodium vivax* transcriptomes reveal stage-specific chloroquine response and differential regulation of male and female gametocytes. Nat Commun. 2019;10:371. 30670687 10.1038/s41467-019-08312-z PMC6342968

[34] Gunalan K, Sá JM, Moraes Barros RR, Anzick SL, Caleon RL, Mershon JP, et al. Transcriptome profiling of *Plasmodium vivax* in *Saimiri* monkeys identifies potential ligands for invasion. Proc Natl Acad Sci U S A. 2019;116:7053. 30872477 10.1073/pnas.1818485116 PMC6452724

[35] Collins WE, Contacos PG, Krotoski WA, Howard WA. Transmission of four Central American strains of Plasmodium vivax from monkey to man. J Parasitol. 1972;58:332–5. 4623380

[36] Boonkaew T, Mongkol W, Prasert S, Paochan P, Yoneda S, Nguitragool W, et al. Transcriptome analysis of *Anopheles dirus* and *Plasmodium vivax* at ookinete and oocyst stages. Acta Trop. 2020;207: 105502. 32320680 10.1016/j.actatropica.2020.105502

[37] Siegel SV, Chappell L, Hostetler JB, Amaratunga C, Suon S, Böhme U, et al. Analysis of Plasmodium vivax schizont transcriptomes from field isolates reveals heterogeneity of expression of genes involved in host-parasite interactions. Sci Rep. 2020;10:16667. 33028892 10.1038/s41598-020-73562-7 PMC7541449

[38] Rangel GW, Clark MA, Kanjee U, Goldberg JM, MacInnis B, José Menezes M, et al. Plasmodium vivax transcriptional profiling of low input cryopreserved isolates through the intraerythrocytic development cycle. PLoS Negl Trop Dis. 2020;14: e0008104. 32119669 10.1371/journal.pntd.0008104 PMC7067476

[39] Bourgard C, Lopes SCP, Lacerda MVG, Albrecht L, Costa FTM. A suitable RNA preparation methodology for whole transcriptome shotgun sequencing harvested from *Plasmodium vivax*-infected patients. Sci Rep. 2021;11:5089. 33658571 10.1038/s41598-021-84607-w PMC7930272

[40] De Meulenaere K, Prajapati SK, Villasis E, Cuypers B, Kattenberg JH, Kasian B, et al. Band 3–mediated Plasmodium vivax invasion is associated with transcriptional variation in PvTRAg genes. Front Cell Infect Microbiol. 2022;12:1011692. 36250048 10.3389/fcimb.2022.1011692 PMC9563252

[41] Little TS, Cunningham DA, Vandomme A, Lopez CT, Amis S, Alder C, et al. Analysis of *pir* gene expression across the *Plasmodium* life cycle. Malar J. 2021;20:1–14. 34823519 10.1186/s12936-021-03979-6 PMC8614022

[42] Gilbert D. Accurate & complete gene construction with EvidentialGene. F1000Res. 2016;5.

[43] Gilbert DG. Genes of the pig, *Sus scrofa*, reconstructed with EvidentialGene. PeerJ. 2019;7: e6374. 30723633 10.7717/peerj.6374 PMC6361002

[44] Simão FA, Waterhouse RM, Ioannidis P, Kriventseva EV, Zdobnov EM. BUSCO: Assessing genome assembly and annotation completeness with single-copy orthologs. Bioinformatics. 2015;31:3210–2. 26059717 10.1093/bioinformatics/btv351

[45] Bushmanova E, Antipov D, Lapidus A, Prjibelski AD. rnaSPAdes: a *de novo* transcriptome assembler and its application to RNA-Seq data. Gigascience. 2019;8:1–13. 10.1093/gigascience/giz100 PMC6736328 31494669

[46] Altschul SF, Madden TL, Schäffer AA, Zhang J, Zhang Z, Miller W, et al. Gapped BLAST and PSI-BLAST:a new generation of protein database search programs. Nucleic Acids Res. 1997;25:3389–402. 9254694 10.1093/nar/25.17.3389 PMC146917

[47] Pain A, Böhme U, Berry AE, Mungall K, Finn RD, Jackson AP, et al. The genome of the simian and human malaria parasite *Plasmodium knowlesi*. Nature. 2008;455:799–803. 18843368 10.1038/nature07306 PMC2656934

[48] Bajic M, Ravishankar S, Sheth M, Rowe LA, Pacheco MA, Patel DS, et al. The first complete genome of the simian malaria parasite Plasmodium brasilianum. Scient Rep. 2022;12:19802. 10.1038/s41598-022-20706-6 PMC9671904 36396703

[49] Pasini EM, Böhme U, Rutledge GG, der Voorberg-Van Wel A, Sanders M, Berriman M, et al. An improved *Plasmodium cynomolgi* genome assembly reveals an unexpected methyltransferase gene expansion. Wellcome Open Res. 2017;2:42. 28748222 10.12688/wellcomeopenres.11864.1 PMC5500898

[50] Tachibana S-I, Sullivan SA, Kawai S, Nakamura S, Kim HR, Goto N, et al. Plasmodium cynomolgi genome sequences provide insight into Plasmodium vivax and the monkey malaria clade. Nat Genet. 2012;44:1051–5. 22863735 10.1038/ng.2375 PMC3759362

[51] Chien J-T, Pakala SB, Geraldo JA, Lapp SA, Humphrey JC, Barnwell JW, et al. High-quality genome assembly and annotation for plasmodium coatneyi, generated using single-molecule real-time pacbio technology. Genome Announc. 2016;4:e00883. 27587810 10.1128/genomeA.00883-16 PMC5009967

[52] Gilabert A, Otto TD, Rutledge GG, Franzon B, Ollomo B, Arnathau C, et al. Plasmodium vivax-like genome sequences shed new insights into Plasmodium vivax biology and evolution. PLoS Biol. 2018;16: e2006035. 30142149 10.1371/journal.pbio.2006035 PMC6130868

[53] Van Dongen S, Abreu-Goodger C. Using MCL to extract clusters from networks. Methods Mol Biol. 2012;804:281–95. 22144159 10.1007/978-1-61779-361-5_15

[54] Zollner GE, Ponsa N, Garman GW, Poudel S, Bell JA, Sattabongkot J, et al. Population dynamics of sporogony for Plasmodium vivax parasites from western Thailand developing within three species of colonized Anopheles mosquitoes. Malar J. 2006;5:1–17. 16887043 10.1186/1475-2875-5-68 PMC1557861

[55] Di Tommaso P, Chatzou M, Floden EW, Barja PP, Palumbo E, Notredame C. Nextflow enables reproducible computational workflows. Nat Biotechnol. 2017;35:316–9. 28398311 10.1038/nbt.3820

[56] Grabherr MG, Haas BJ, Yassour M, Levin JZ, Thompson DA, Amit I, et al. Full-length transcriptome assembly from RNA-Seq data without a reference genome. Nat Biotechnol. 2011;29:644–52. 21572440 10.1038/nbt.1883 PMC3571712

[57] Bankevich A, Nurk S, Antipov D, Gurevich AA, Dvorkin M, Kulikov AS, et al. SPAdes: A new genome assembly algorithm and its applications to single-cell sequencing. J Comput Biol. 2012;19:455–77. 22506599 10.1089/cmb.2012.0021 PMC3342519

[58] Mamrot J, Legaie R, Ellery SJ, Wilson T, Seemann T, Powell DR, et al. *De novo* transcriptome assembly for the spiny mouse (*Acomys cahirinus*). Scient Rep. 2017;7:1. 10.1038/s41598-017-09334-7 PMC5566366 28827620

[59] Chopra R, Burow G, Farmer A, Mudge J, Simpson CE, Burow MD. Comparisons of *de novo* transcriptome assemblers in diploid and polyploid species using peanut (*Arachis spp.*) RNA-Seq data. PLoS One. 2014;9;e115055. 25551607 10.1371/journal.pone.0115055 PMC4281230

[60] Amin S, Prentis PJ, Gilding EK, Pavasovic A. Assembly and annotation of a non-model gastropod (*Nerita melanotragus*) transcriptome: A comparison of *de novo* assemblers. BMC Res Notes. 2014;7:1–8. 25084827 10.1186/1756-0500-7-488 PMC4124492

[61] Francis WR, Christianson LM, Kiko R, Powers ML, Shaner NC, Haddock SHD. A comparison across non-model animals suggests an optimal sequencing depth for *de novo* transcriptome assembly. BMC Genomics. 2013;14:1–12. 23496952 10.1186/1471-2164-14-167 PMC3655071

[62] Madritsch S, Burg A, Sehr EM. Comparing *de novo* transcriptome assembly tools in di- and autotetraploid non-model plant species. BMC Bioinformatics. 2021;22:1–17. 33752598 10.1186/s12859-021-04078-8 PMC7986043

[63] Yahav T, Privman E. A comparative analysis of methods for *de novo* assembly of hymenopteran genomes using either haploid or diploid samples. Sci Rep. 2019;9:1–10. 31019201 10.1038/s41598-019-42795-6 PMC6482151

[64] Rana SB, Zadlock FJ, Zhang Z, Murphy WR, Bentivegna CS. Comparison of *de novo* transcriptome assemblers and k-mer strategies using the killifish. Fundulus heteroclitus PLoS One. 2016;11: e0153104. 27054874 10.1371/journal.pone.0153104 PMC4824410

[65] Hölzer M, Marz M. *De novo* transcriptome assembly: A comprehensive cross-species comparison of short-read RNA-Seq assemblers. Gigascience. 2019;8:1–16. 10.1093/gigascience/giz039 PMC6511074 31077315

[66] Chang Z, Li G, Liu J, Zhang Y, Ashby C, Liu D, et al. Bridger: A new framework for *de novo* transcriptome assembly using RNA-seq data. Genome Biol. 2015;16:1–10. 25723335 10.1186/s13059-015-0596-2 PMC4342890

[67] Birol I, Jackman SD, Nielsen CB, Qian JQ, Varhol R, Stazyk G, et al. De novo transcriptome assembly with ABySS. Bioinformatics. 2009;25:2872–7. 19528083 10.1093/bioinformatics/btp367

[68] Xie Y, Wu G, Tang J, Luo R, Patterson J, Liu S, et al. SOAPdenovo-Trans: *de novo* transcriptome assembly with short RNA-Seq reads. Bioinformatics. 2014;30:1660–6. 24532719 10.1093/bioinformatics/btu077

[69] Pasini EM, Braks JA, Fonager J, Klop O, Aime E, Spaccapelo R, et al. Proteomic and genetic analyses demonstrate that *Plasmodium berghei* blood stages export a large and diverse repertoire of proteins. Mol Cell Proteomics. 2013;12:426–48. 23197789 10.1074/mcp.M112.021238 PMC3567864

[70] Howick VM, Russell AJC, Andrews T, Heaton H, Reid AJ, Natarajan K, et al. The Malaria Cell Atlas: Single parasite transcriptomes across the complete *Plasmodium* life cycle. Science. 1979;2019(eaaw365):2619. 10.1126/science.aaw2619 PMC7056351 31439762

[71] Cunningham DA, Reid AJ, Hosking C, Deroost K, Tumwine-Downey I, Sanders M, et al. Identification of gametocyte-associated pir genes in the rodent malaria parasite, Plasmodium chabaudi chabaudi AS. BMC Res Notes. 2023;16:56. 37076932 10.1186/s13104-023-06322-1 PMC10114299

[72] Hoo R, Zhu L, Amaladoss A, Mok S, Natalang O, Lapp SA, et al. Integrated analysis of the *Plasmodium* species transcriptome. EBioMedicine. 2016;7:255–66. 27322479 10.1016/j.ebiom.2016.04.011 PMC4909483

[73] Bass CC, Johns FM. The cultivation of malarial plasmodia (plasmodium vivax and plasmodium falciparum) in vitro. J Exp Med. 1912;16:567–79. 19867597 10.1084/jem.16.4.567 PMC2124976

[74] Noulin F, Borlon C, Van Den Abbeele J, D’Alessandro U, Erhart A. 1912–2012: A century of research on Plasmodium vivax in vitro culture. Trends Parasitol. 2013;29:286–94. 23623759 10.1016/j.pt.2013.03.012

[75] Thomson JG, Thomson D, Fantham HB. The cultivation of one generation of benign tertian malarial parasites (plasmodium vivax) in vitro, by bass’s method. Ann Trop Med Parasitol. 1913;7:153–64.

[76] Hupalo DN, Luo Z, Melnikov A, Sutton PL, Rogov P, Escalante A, et al. Population genomics studies identify signatures of global dispersal and drug resistance in Plasmodium vivax. Nat Genet. 2016;48:953–8. 27348298 10.1038/ng.3588 PMC5347536

[77] Imwong M, Nair S, Pukrittayakamee S, Sudimack D, Williams JT, Mayxay M, et al. Contrasting genetic structure in Plasmodium vivax populations from Asia and South America. Int J Parasitol. 2007;37:1013–22. 17442318 10.1016/j.ijpara.2007.02.010

[78] Mu J, Joy DA, Duan J, Huang Y, Carlton J, Walker J, et al. Host switch leads to emergence of plasmodium vivax malaria in humans. Mol Biol Evol. 2005;22:1686–93. 15858201 10.1093/molbev/msi160

[79] Daron J, Boissière A, Boundenga L, Ngoubangoye B, Houze S, Arnathau C, et al. Population genomic evidence of Plasmodium vivax Southeast Asian origin. Sci Adv. 2021;7:eabc3713. 33910900 10.1126/sciadv.abc3713 PMC8081369

[80] Prajapati SK, Joshi H, Carlton JM, Rizvi MA. Neutral polymorphisms in putative housekeeping genes and tandem repeats unravels the population genetics and evolutionary history of plasmodium vivax in India. PLoS Negl Trop Dis. 2013;7:e2425. 24069480 10.1371/journal.pntd.0002425 PMC3777877

[81] Kepple D, Ford CT, Williams J, Abagero B, Li S, Popovici J, et al. Comparative transcriptomics reveal differential gene expression among Plasmodium vivax geographical isolates and implications on erythrocyte invasion mechanisms. PLoS Negl Trop Dis. 2024;18: e0011926. 38285730 10.1371/journal.pntd.0011926 PMC10901308

[82] De Meulenaere K, Cuypers B, Gamboa D, Laukens K, Rosanas-Urgell A. A new Plasmodium vivax reference genome for South American isolates. BMC Genomics. 2023;24:1–14. 37821878 10.1186/s12864-023-09707-5 PMC10568799

[83] Callejas-Hernández F, Nikulkova M, Adamski N, Yan G, Yewhalaw D, Carlton JM. Assembled genome of an Ethiopian Plasmodium vivax isolate generated using GridION long-read technology. Microbiol Resour Announc. 2024;13:e00590-e624. 39283158 10.1128/mra.00590-24 PMC11465822

[84] Brashear AM, Huckaby AC, Fan Q, Dillard LJ, Hu Y, Li Y, et al. New *Plasmodium vivax* Genomes From the China-Myanmar Border. Front Microbiol. 2020;11:1930. 32849480 10.3389/fmicb.2020.01930 PMC7432439

[85] Kim D, Langmead B, Salzberg SL. HISAT: a fast spliced aligner with low memory requirements. Nat Methods. 2015;12:357–60. 25751142 10.1038/nmeth.3317 PMC4655817

[86] Schneider VA, Graves-Lindsay T, Howe K, Bouk N, Chen HC, Kitts PA, et al. Evaluation of GRCh38 and de novo haploid genome assemblies demonstrates the enduring quality of the reference assembly. Genome Res. 2017;27:849–64. 28396521 10.1101/gr.213611.116 PMC5411779

[87] Amos B, Aurrecoechea C, Barba M, Barreto A, Basenko EY, Bażant W, et al. VEuPathDB: the eukaryotic pathogen, vector and host bioinformatics resource center. Nucleic Acids Res. 2022;50:D898-911. 34718728 10.1093/nar/gkab929 PMC8728164

[88] Neafsey DE, Waterhouse RM, Abai MR, Aganezov SS, Alekseyev MA, Allen JE, et al. Mosquito genomics. Highly evolvable malaria vectors: the genomes of 16 Anopheles mosquitoes. Science. 1979;2015(347):1258522. 10.1126/science.1258522 PMC4380271 25554792

[89] Marinotti O, Cerqueira GC, de Almeida LGP, Ferro MIT, da Silva Loreto EL, Zaha A, et al. The genome of Anopheles darlingi, the main neotropical malaria vector. Nucleic Acids Res. 2013;41:7387–400. 23761445 10.1093/nar/gkt484 PMC3753621

[90] Chiou KL, Pozzi L, Lynch Alfaro JW, di Fiore A. Pleistocene diversification of living squirrel monkeys (*Saimiri spp*) inferred from complete mitochondrial genome sequences. Mol Phylogenet Evol. 2011;59:736–45. 21443955 10.1016/j.ympev.2011.03.025

[91] Babb PL, Fernandez-Duque E, Baiduc CA, Gagneux P, Evans S, Schurr TG. mtDNA diversity in azara’s owl monkeys (*Aotus azarai azarai*) of the Argentinean Chaco. Am J Phys Anthropol. 2011;146:209–24. 21826638 10.1002/ajpa.21567

[92] Kopylova E, Noé L, Touzet H. SortMeRNA: fast and accurate filtering of ribosomal RNAs in metatranscriptomic data. Bioinformatics. 2012;28:3211–7. 23071270 10.1093/bioinformatics/bts611

[93] Manni M, Berkeley MR, Seppey M, Simão FA, Zdobnov EM. BUSCO Update: Novel and Streamlined Workflows along with Broader and Deeper Phylogenetic Coverage for Scoring of Eukaryotic, Prokaryotic, and Viral Genomes. Mol Biol Evol. 2021;38:4647–54. 34320186 10.1093/molbev/msab199 PMC8476166

[94] Mistry J, Chuguransky S, Williams L, Qureshi M, Salazar GA, Sonnhammer ELL, et al. Pfam: The protein families database in 2021. Nucleic Acids Res. 2021;49:D412–9. 33125078 10.1093/nar/gkaa913 PMC7779014

[95] Eddy SR. Accelerated Profile HMM Searches. PLoS Comput Biol. 2011;7:1002195. 10.1371/journal.pcbi.1002195 PMC3197634 22039361

[96] Patro R, Duggal G, Love MI, Irizarry RA, Kingsford C. Salmon provides fast and bias-aware quantification of transcript expression. Nat Methods. 2017;14:417–9. 28263959 10.1038/nmeth.4197 PMC5600148

[97] Liao Y, Smyth GK, Shi W. The R package Rsubread is easier, faster, cheaper and better for alignment and quantification of RNA sequencing reads. Nucleic Acids Res. 2019;47:e47–e47. 30783653 10.1093/nar/gkz114 PMC6486549

[98] Bastian M, Heymann S, Jacomy M. Gephi: An Open Source Software for Exploring and Manipulating Networks. 2009.

[99] Martin S, Brown WM, Klavans R, Boyack KW. OpenOrd: an open-source toolbox for large graph layout. In: Visualization and Data Analysis 2011. SPIE; 2011. p. 786806.

[100] Wickham H. stringr: Simple, Consistent Wrappers for Common String Operations. R package version 1.4.0. 2019.

[101] Wickham H, Romain F, Henry L, Kirill M. dplyr: A Grammar of Data Manipulation. R package version 0.8.1. 2019. https://cran.r-project.org/package=dplyr.

[102] Dowle M, Srinivasan A. data.table: Extension of `data.frame`. R package version. 2019.

[103] Wickham H. ggplot2: Elegant Graphics for Data Analysis. New York: Springer-Verlag; 2016.

[104] Gu Z, Eils R, Schlesner M. Complex heatmaps reveal patterns and correlations in multidimensional genomic data. Bioinformatics. 2016;32:2847–9. 27207943 10.1093/bioinformatics/btw313

[105] Gu Z, Gu L, Eils R, Schlesner M, Brors B. Circlize implements and enhances circular visualization in R. Bioinformatics. 2014;30:2811–2. 24930139 10.1093/bioinformatics/btu393

[106] Garnier S. viridis: Default Color Maps from “matplotlib”. R package version 0.5.1. 2018.

[107] R Core Team. R: A language and environment for statistical computing. R Foundation for Statistical Computing, Vienna, Austria. 2018. https://www.r-project.org/.

