# Supplementary figures and images for: De novo assembly of plasmodium interspersed repeat (pir) genes from Plasmodium vivax RNAseq data suggests geographic conservation of sub-family transcription
Source: BMC Genomics. 2025 May 29;26:544. doi: 10.1186/s12864-025-11752-1 (PMC12121038; doi:10.1186/s12864-025-11752-1)

A.

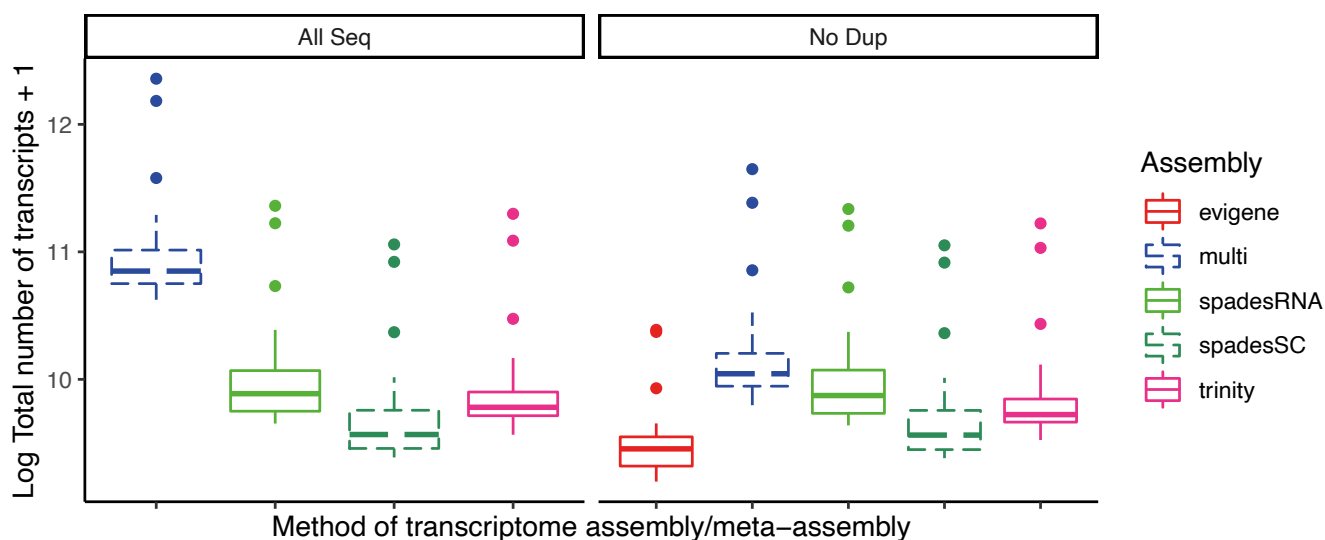

B.

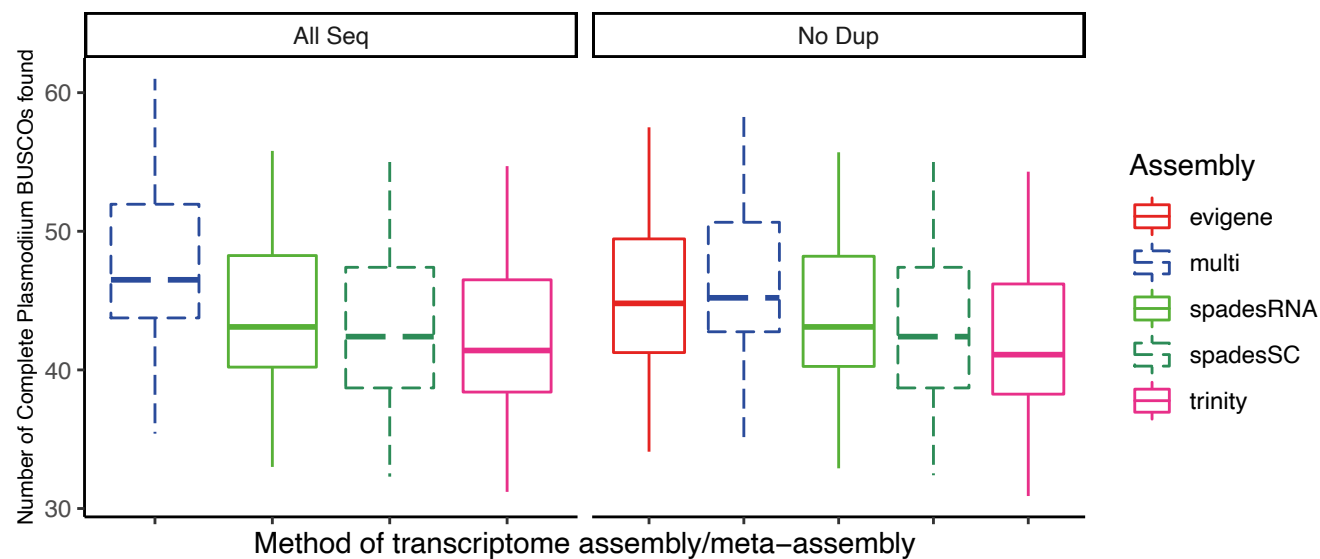

C.

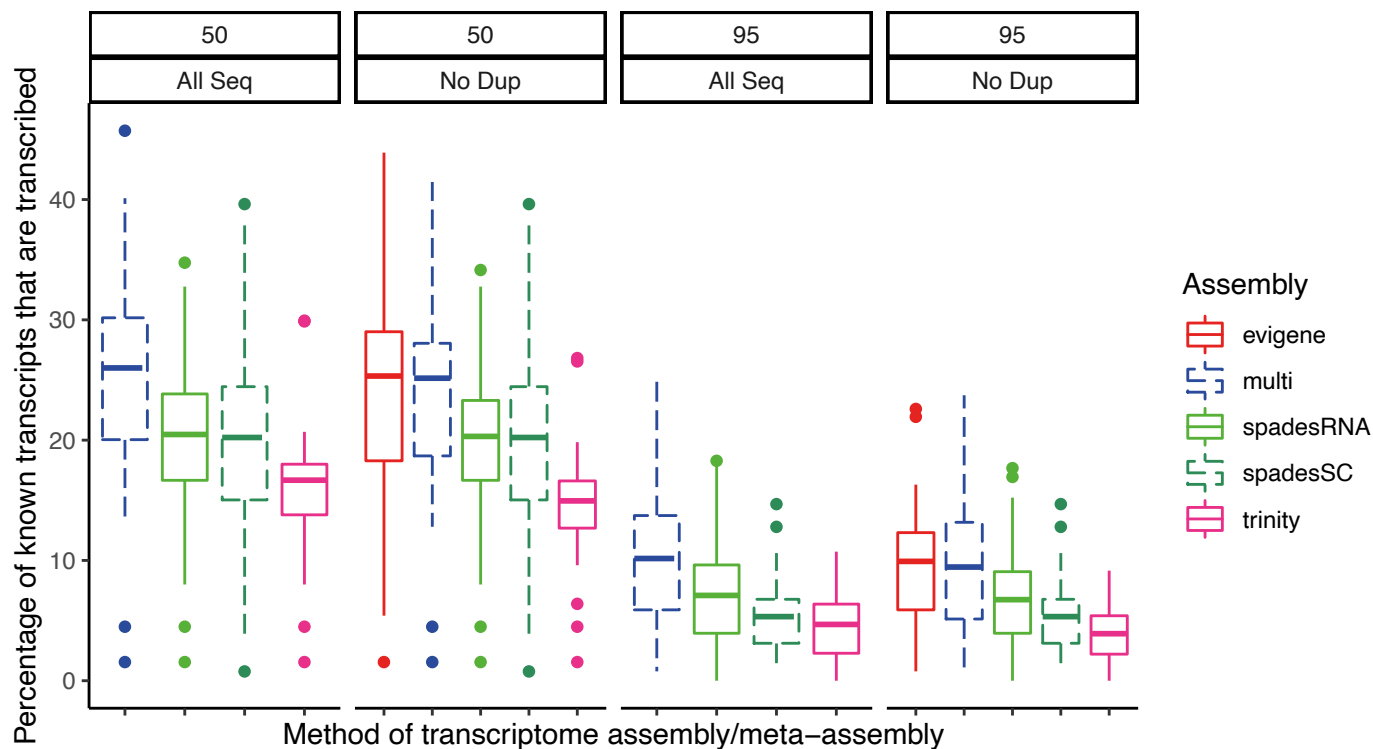

Supplement: Supplementary file 5 — Additional file 5. Supplementary Figure 1. [file 12864_2025_11752_MOESM5_ESM.pdf]

Percentage  
reference  
*pir*  
coverage

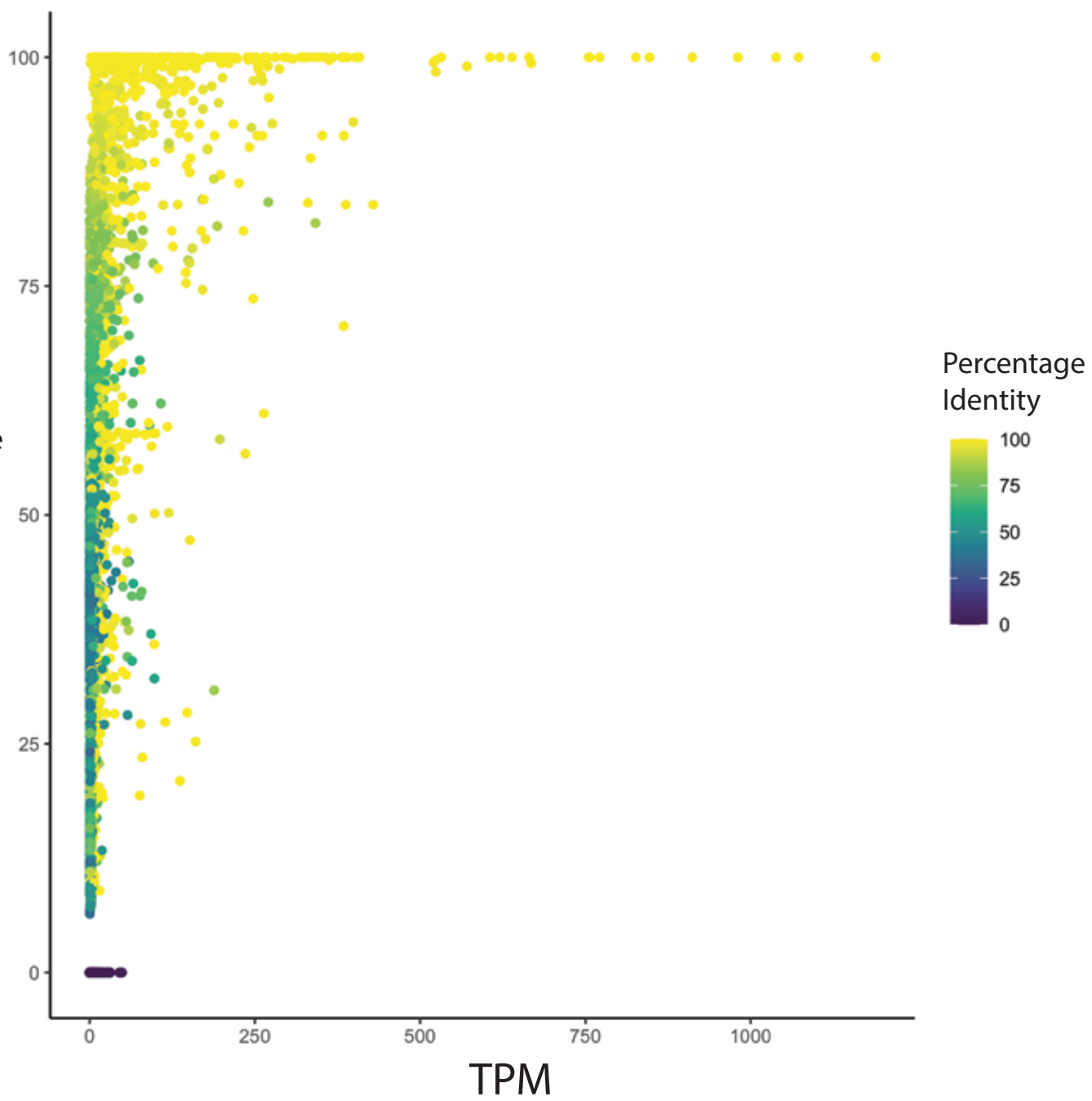

Supplement: Supplementary file 6 — Additional file 6. Supplementary Figure 2 [file 12864_2025_11752_MOESM6_ESM.pdf]

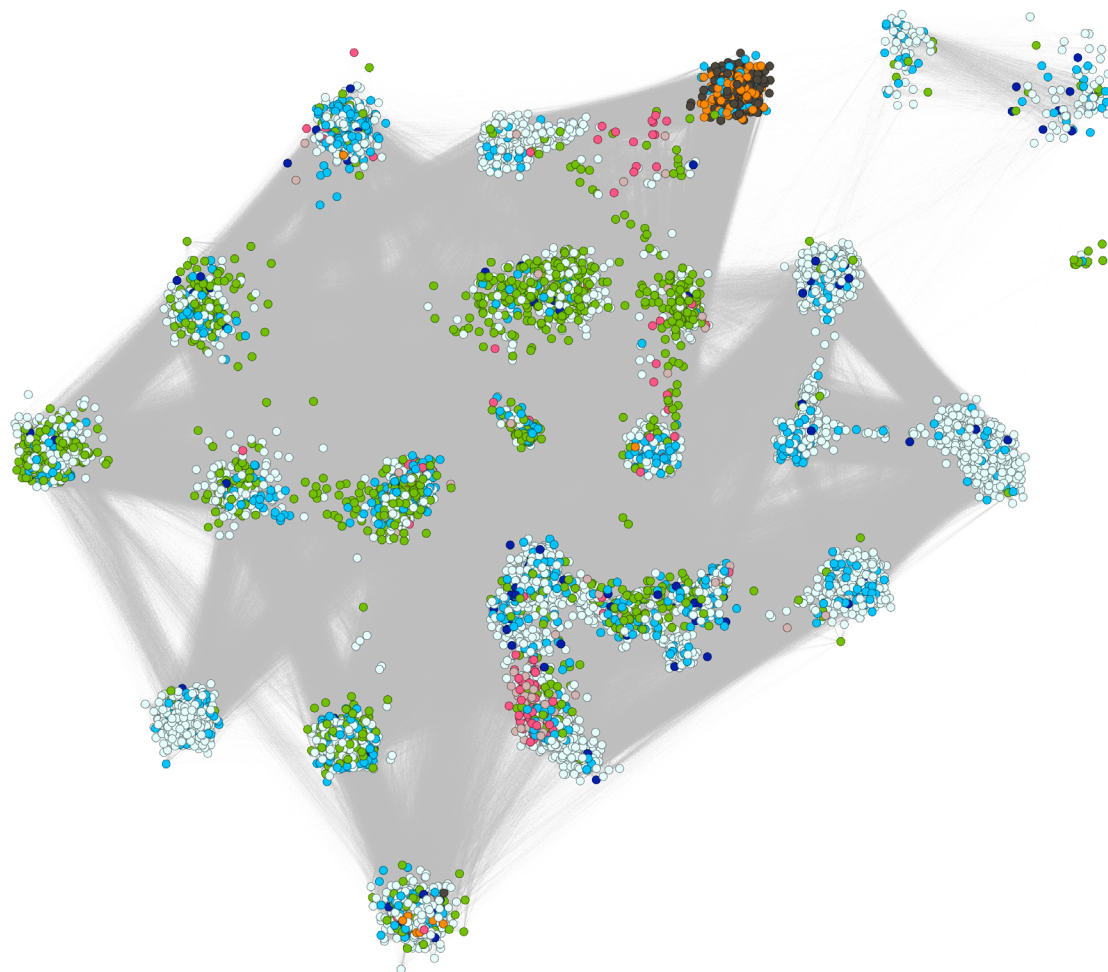

- vivax*
- ovale*
- cyno*
- coat*
- knowl*
- malar*
- vvx-like*
- brasl*

Supplement: Supplementary file 7 — Additional file 7. Supplementary Figure 3 [file 12864_2025_11752_MOESM7_ESM.pdf]

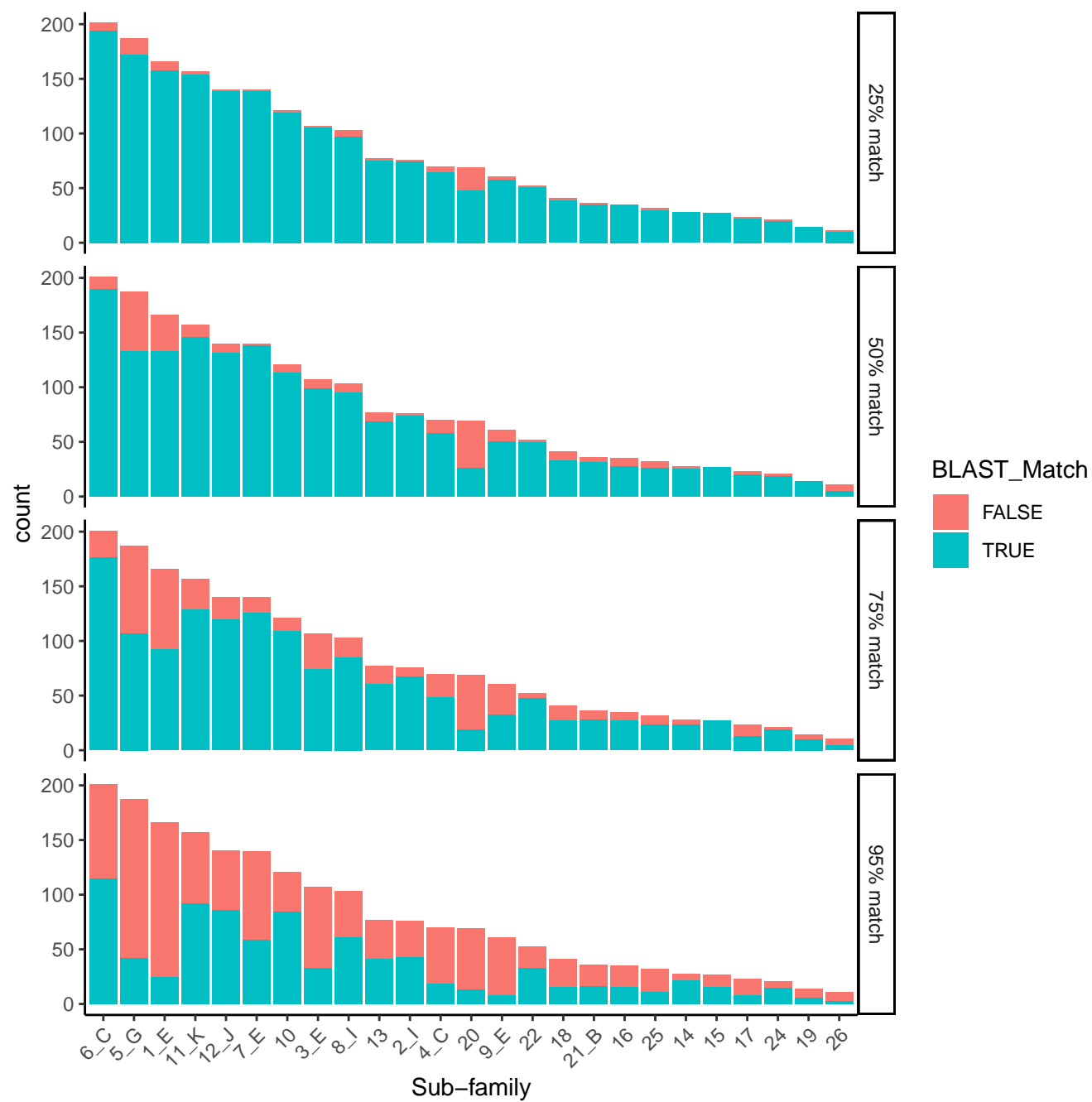

Supplement: Supplementary file 8 — Additional file 8. Supplementary Figure 4 [file 12864_2025_11752_MOESM8_ESM.pdf]

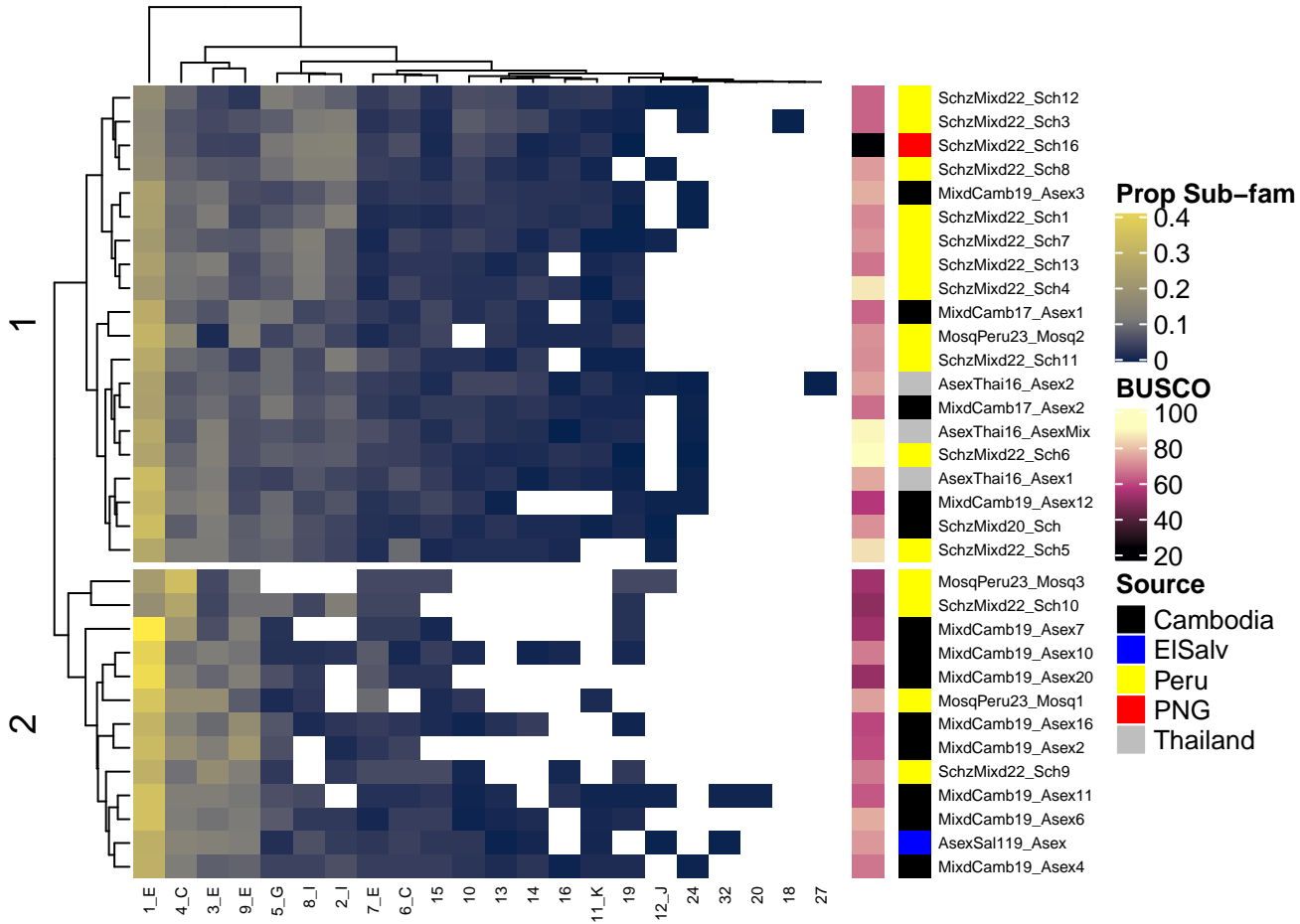

Supplement: Supplementary file 9 — Additional file 9. Supplementary Figure 5 [file 12864_2025_11752_MOESM9_ESM.pdf]

## AsexThai16\_SMRU1

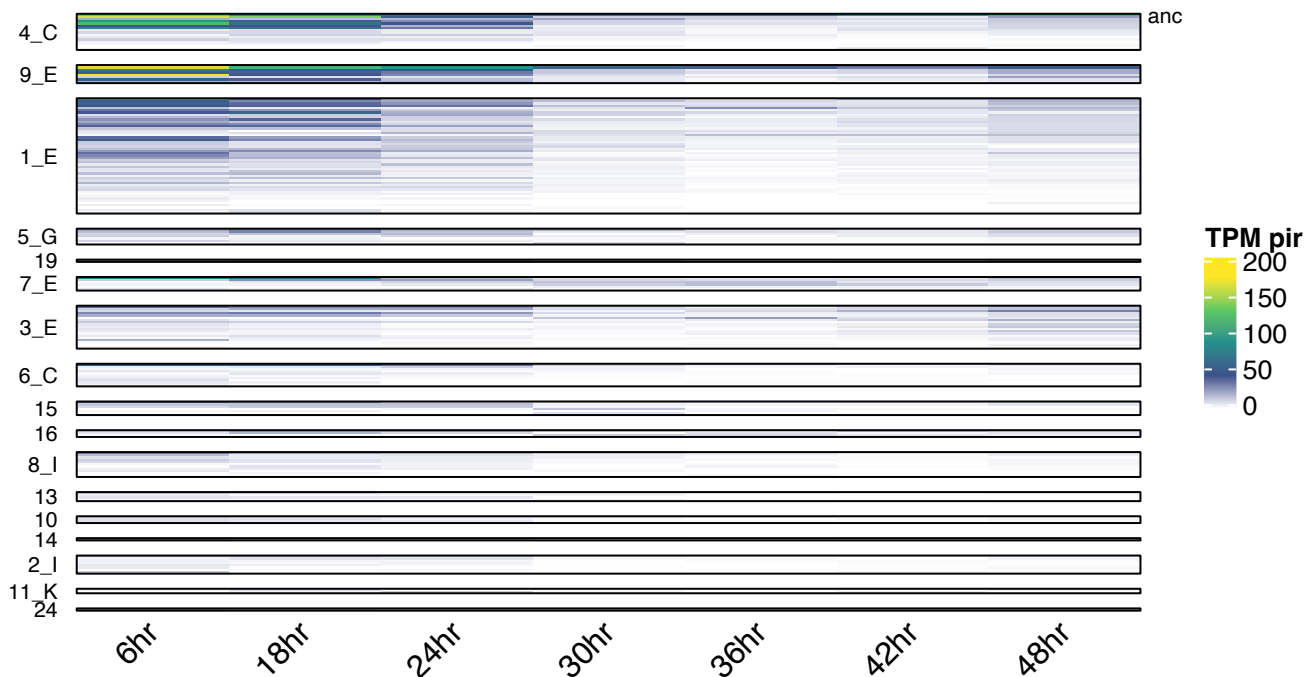

## AsexThai16\_SMRU2

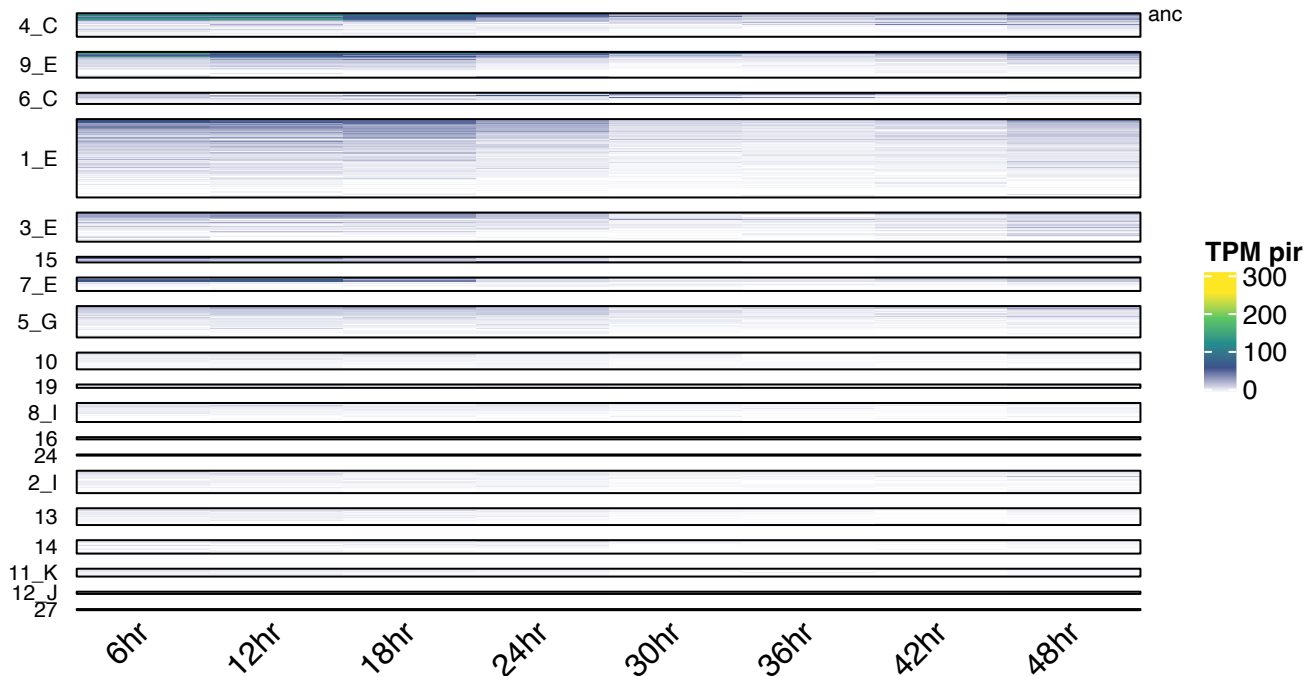

Supplement: Supplementary file 10 — Additional file 10. Supplementary Figure 6 [file 12864_2025_11752_MOESM10_ESM.pdf]

# AsexSal119\_Sal1

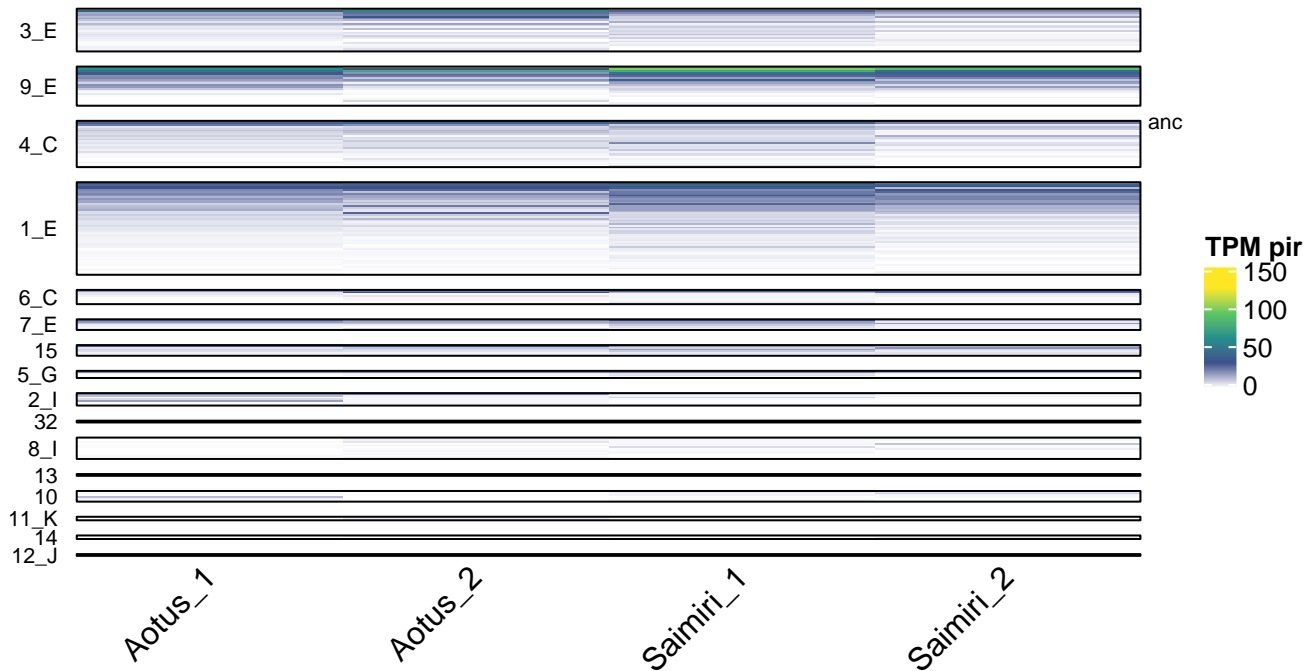

Supplement: Supplementary file 11 — Additional file 11. Supplementary Figure 7 [file 12864_2025_11752_MOESM11_ESM.pdf]

# MosqPeru23\_P6

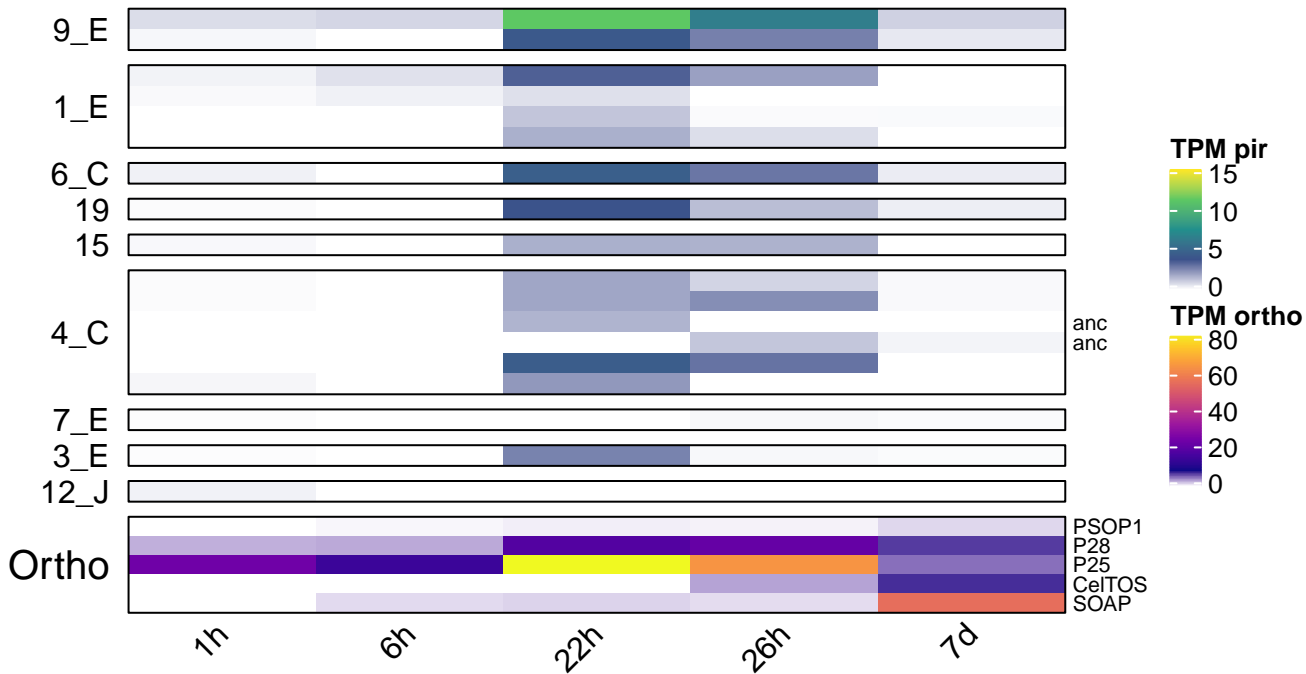

Supplement: Supplementary file 12 — Additional file 12. Supplementary Figure 8 [file 12864_2025_11752_MOESM12_ESM.pdf]
